# Single-cell atlas of cervical organoids uncovers epithelial immune heterogeneity and intercellular cross-talk during Chlamydia infection

**DOI:** 10.1126/sciadv.ady1640

**Published:** 2025-10-03

**Authors:** Pon Ganish Prakash, Naveen Kumar, Stefanie Koster, Christian Wentland, Jayabhuvaneshwari Dhanraj, Rajendra Kumar Gurumuthy, Cindrilla Chumduri

**Affiliations:** ^1^ Department of Microbiology, University of Würzburg, Würzburg, Germany.; ^2^ Infection, Carcinogenesis and Regeneration, Medical Biotechnology Section, Department of Biological and Chemical Engineering, Aarhus University, Aarhus, Denmark.; ^3^ Department of Molecular Biology, Max Planck Institute for Infection Biology, Berlin, Germany.

## Abstract

The uterine cervix is a critical mucosal interface that balances immune defense and reproductive function, yet how its distinct epithelial compartments coordinate responses to infection remains unclear. Here, we integrate patient-derived three-dimensional cervical organoids, single-cell transcriptomics, and native tissue analysis to construct a high-resolution atlas of epithelial cell diversity and immune dynamics during *Chlamydia trachomatis* infection. We demonstrate that cervical organoids closely resemble native tissue at transcriptional and cellular levels, identifying epithelial subtypes with region-specific immune specializations. Upon infection, ectocervical epithelia reinforce barrier integrity, whereas endocervical epithelia, particularly uninfected bystander cells, exhibit extensive transcriptional reprogramming characterized by robust interferon activation, antigen presentation, and antimicrobial defense. Infection profoundly reshapes epithelial intercellular communication, positioning bystander cells as major contributors to signaling networks coordinating immune responses and tissue regeneration. Our findings highlight a sophisticated epithelial-intrinsic immune network critical for cervical mucosal defense and establish a physiologically relevant platform for studying host-pathogen interactions and guiding targeted mucosal therapies against reproductive tract infections and pathologies.

## Teaser

Organoids and single-cell analysis uncover diverse cervical epithelial immune strategies to fight infection.

## INTRODUCTION

Epithelial cells form the frontline of defense across human organ systems, playing pivotal roles in tissue homeostasis, immune surveillance, and pathogen defense (*1*, *2*). In the female reproductive tract (FRT), the cervical epithelium serves as both a structural and immunological barrier that simultaneously supports reproductive functions and protects against microbial invasion (*3*). The uterine cervix, located at the interface of the upper and lower FRT, comprises two distinct epithelial compartments, the stratified squamous epithelium of the ectocervix and the columnar, mucus-producing epithelium of the endocervix. These regions differ in both function and developmental origin and converge at the squamo-columnar junction, a site of active remodeling and high susceptibility to metaplasia and oncogenic transformation (*4*–*6*).

The cervix is a primary entry point for sexually transmitted pathogens, including *Chlamydia trachomatis* (Chlamydia) and high-risk human papillomaviruses (HPVs). Chlamydia, the most prevalent bacterial sexually transmitted infection worldwide, establishes persistent infections that are often asymptomatic yet can lead to serious reproductive complications such as pelvic inflammatory disease, infertility, and ectopic pregnancy (*7*, *8*). Chronic Chlamydia infection is also implicated in modulating epithelial cell fate, compromising genomic surveillance, and promoting HPV-associated carcinogenesis (*9*, *10*). Despite the clear relevance of epithelial cell responses in cervical infections, the region- and cell type–specific immune defense mechanisms of the ecto- and endocervical epithelia remain incompletely defined.

Traditional in vitro models, including immortalized cervical cell lines, have provided foundational insights into Chlamydia pathogenesis but lack the structural, cellular, and immunological complexity of the human cervix (*11*, *12*). Patient-derived three-dimensional (3D) organoid models now offer a powerful alternative. These self-organizing structures preserve tissue-specific lineage fidelity, cellular heterogeneity, and structural polarization, offering unprecedented physiological relevance for infection biology (*13*, *14*). Recent studies have used cervical organoids to explore HPV and Chlamydia coinfections and epithelial transformation (*15*), yet how distinct epithelial cell types coordinate immune defense and intercellular signaling during infection remains poorly understood.

Single-cell RNA sequencing (scRNA-seq) has revolutionized our ability to dissect epithelial complexity and cellular responses at high resolution. When combined with 3D organoid systems, it enables comprehensive mapping of epithelial differentiation trajectories, infection-induced remodeling, and intercellular communication networks (*16*, *17*). This integrative approach provides powerful insights into epithelial-intrinsic immunity and the coordinated tissue-level organization of mucosal defense.

In this study, we leverage scRNA-seq and patient-derived ecto- and endocervical organoids to construct a high-resolution cellular atlas of cervical epithelial heterogeneity during Chlamydia infection. We validate the transcriptional fidelity of cervical organoids against native tissue and uncover distinct immune specializations of squamous and columnar epithelia. Upon infection, we observe profound transcriptional reprogramming in endocervical bystander cells, including interferon (IFN) activation, antimicrobial responses, and activation of antigen-presentation pathways. In contrast, ectocervical cells prioritize structural maintenance and regeneration, exhibiting restrained immune activation. Using ligand-receptor (L-R) interaction modeling, we show that infection rewires epithelial communication networks, with bystander cells emerging as key epithelial signaling node for immune coordination and regenerative signaling. Together, our work reveals a compartmentalized yet integrated epithelial defense architecture and offers a powerful platform to advance mechanistic understanding of host-pathogen interactions and epithelial remodeling in the FRT, with future potential to inform targeted therapeutic strategies.

## RESULTS

### Integrative scRNA-seq analysis of patient-derived cervical organoids and native tissue

**Fig. 1.**
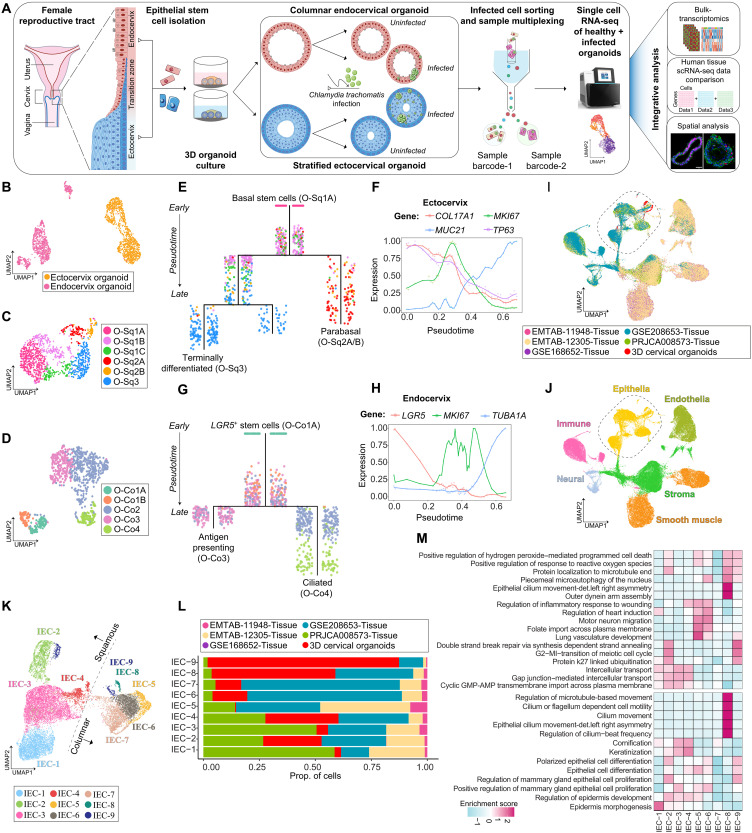
Experimental setup and single-cell characterization of the cervical epithelial landscape using patient-derived 3D organoids. (**A**) Schematic of the experimental workflow showing the generation of patient-derived uninfected and Chlamydia-infected ecto- and endocervical organoids, followed by scRNA-seq. (**B**) Uniform Manifold Approximation and Projection (UMAP) of ecto- and endocervical epithelial cell clusters. Each dot represents a single-cell color-coded by tissue type. (**C** and **D**) UMAP projections highlighting squamous (C) and columnar (D) epithelial subclusters derived from ecto- and endocervical organoids, respectively, with cells colored by cluster. (**E**) URD differentiation tree of ectocervical squamous epithelial cells; each dot represents a single cell, colored by subcluster. Cells ordered on the basis of pseudotime values starting from early (top) to late (bottom). (**F**) Gene expression dynamics of selected squamous markers along the pseudotime; lines represent expression trend of a particular gene. (**G**) Differentiation trajectory of endocervical columnar epithelial cells, colored by subcluster and ordered by pseudotime (early to late). (**H**) Expression dynamics of key columnar epithelial marker genes along pseudotime. (**I**) UMAP visualization of organoid and tissue-derived cell clusters after data integration; cells are color-coded on the basis of their dataset of origin. (**J**) UMAP shows six major cell populations across the integrated dataset. (**K**) UMAP depicting nine epithelial subclusters identified postintegration, with cells color-coded by cluster. (**L**) Bar plot depicting the epithelial cell proportions from different datasets across integrated subclusters. (**M**) Heatmap showing gene set enrichment scores for GO biological processes across integrated epithelial clusters; scale bar denotes the *z*-scored enrichment values ranging from high (deep pink) to low (blue).

To investigate host-pathogen interactions and epithelial cellular dynamics in the cervix, we combined patient-derived cervical organoids with scRNA-seq. Organoids were generated from adult epithelial stem cells isolated from ecto- and endocervical tissues of healthy donors, expanded in lineage-specific cultures, and subsequently infected with green fluorescent protein (GFP)–labeled Chlamydia. Infected and uninfected cells were sorted using fluorescence-activated cell sorting (FACS), and high-throughput scRNA-seq was performed using the 10X Genomics platform, capturing epithelial states in both homeostasis and infection (Fig. 1A). After quality control, 4985 high-quality single cells were retained for further analysis.

We first examined uninfected organoids to explore epithelial heterogeneity under homeostatic conditions. Unsupervised clustering segregated ecto- and endocervical cells into two transcriptionally distinct clusters, reflecting their lineage specificity and functional divergence (Fig. 1B). These findings were further corroborated by microarray analysis (fig. S1, A and B, and table S1). Gene ontology (GO) analysis of differentially expressed genes revealed that ectocervical squamous (Sq) epithelial cells were enriched for keratinocyte differentiation and cornification, whereas endocervical columnar (Co) epithelium showed enrichment for cilium assembly and organization (fig. S1C).

Subclustering of the scRNA-seq data revealed six squamous epithelial subtypes from ectocervical organoids (O-Sq1A, O-Sq1B, O-Sq1C, O-Sq2A, O-Sq2B, and O-Sq3) and five columnar epithelial subpopulations from endocervical organoids (O-Co1A, O-Co1B, O-Co2, O-Co3, and O-Co4) (Fig. 1, C and D, and fig. S1D). Within the squamous lineage, O-Sq1A cells exhibited a stem-like basal phenotype, marked by *COL17A1*, *TP63*, and *ODC1*. O-Sq1B cells were highly proliferative, expressing *CDK1* and *MKI67*, while O-Sq1C cells displayed early differentiation markers, including *KRT14* and *KRT6A*. Parabasal populations (O-Sq2A and O-Sq2B) expressed *SOX4* and *FOSB* (*18*), while terminally differentiated O-Sq3 cells were enriched for *CRNN* and *KRT13*, reflecting epithelial maturation (fig. S1, E to G).

Pseudotime analysis revealed two distinct differentiation trajectories originating from O-Sq1A basal stem-like cells: one leading to parabasal cells (O-Sq2A and O-Sq2B) and the other toward terminally differentiated squamous cells (O-Sq3) (Fig. 1E and fig. S1H). As cells progressed along these trajectories, expression of stem cell markers (*COL17A1* and *TP63*) declined, while differentiation markers such as *MUC21* increased, indicating maturation. Notably, *MKI67* expression peaked mid-trajectory, marking a transient proliferative phase before cells reached terminal differentiation (Fig. 1F).

In the endocervical epithelium, subclusters O-Co1A and O-Co1B exhibited distinct molecular profiles, with O-Co1A cells expressing *LGR5*, a key stem cell marker essential for glandular epithelial regeneration (*19*), whereas O-Co1B cells predominantly expressed *MUC5B* and *MUC5AC*, mucins involved in pathogen defense (*20*, *21*). Despite their differences, both subclusters expressed *SLC26A2*, *GUCY1A1*, and *DMBT1*, genes associated with epithelial homeostasis and protection against pathogens (*22*, *23*). O-Co2 cells displayed high *MKI67* and *HMGB2* expression, indicating active proliferation, while O-Co3 cells expressed both class I [human leukocyte antigen A (*HLA-A*) and *HLA-B*] and class II (*HLA-DQA1* and *HLA-DRA*) major histocompatibility complex (MHC) genes, suggesting a potential nonprofessional antigen-presenting cell (np-APC) phenotype (*24*, *25*). O-Co4 cells, representing highly differentiated ciliated cells, exhibited strong *TUBA1A* and *FOXJ1* expression (fig. S1, I to K). Developmental trajectory analysis revealed two differentiation pathways originating from *LGR5*^+^ O-Co1A stem cells: one leading to np-APCs (O-Co3) and the other to ciliated cells (O-Co4) (Fig. 1G and fig. S1L). An inverse correlation between *LGR5* and *TUBA1A* expression indicated a progressive shift from stem-like states to terminally differentiated cell types, with *MKI67* expression peaking at the bifurcation point, signifying a proliferative intermediate state (Fig. 1H). Differential expression analysis identified gene signatures unique to squamous and columnar subclusters (fig. S1, F and J, and tables S2 and S3), and cell-type proportion analysis quantified the relative abundance of each epithelial subpopulation within organoid cultures (fig. S1M).

To assess whether patient-derived organoids faithfully recapitulate in vivo transcriptional profiles, we integrated five publicly available human cervical tissue scRNA-seq datasets (*26*–*30*). Following batch correction, cell clustering was driven by transcriptional identity rather than dataset origin, highlighting robust integration (Fig. 1I and fig. S2A). Unsupervised clustering identified six major cell types across datasets, including epithelial, stromal, smooth muscle, immune, neural, and endothelial cells (Fig. 1J and fig. S2B). Notably, epithelial cells from organoids clustered seamlessly with native tissue, confirming their transcriptional fidelity (Fig. 1, I and J).

Next, reclustering of the integrated epithelial cell (IEC) population revealed nine transcriptionally distinct subclusters (IEC-1 to IEC-9) (Fig. 1K and fig. S2C), corresponding to squamous and columnar differentiation states (fig. S2D). IEC-1 to IEC-4 represented squamous epithelial subsets, including basal (IEC-1), proliferative (IEC-2), parabasal (IEC-3), and terminally differentiated (IEC-4) cells. IEC-5 to IEC-9 comprised columnar subtypes, including early progenitors (IEC-5 and IEC-6), np-APCs (IEC-7), and ciliated cells (IEC-8). IEC-9, a columnar epithelial population, included a subset of cells that clustered adjacent to proliferative squamous cells (IEC-2), suggesting a shared proliferative transcriptional signature.

We then examined the proportion of epithelial subclusters (IEC-1 to IEC-9) across both organoid and tissue-derived datasets. Notably, patient-derived 3D organoids contributed to all nine epithelial subpopulations, demonstrating their ability to recapitulate the cellular diversity of native cervical tissue (Fig. 1L). To functionally annotate these epithelial subtypes, we performed gene set enrichment analysis (GSEA). Squamous subclusters (IEC-1 to IEC-4) were enriched for epidermis morphogenesis and cornification, while proliferative subclusters (IEC-2 and IEC-9) showed enrichment for G_2_-M cell cycle transition. The ciliated subpopulation (IEC-8) was notably enriched for cilium motility and beat frequency, confirming its identity (Fig. 1M). These findings emphasize the organoid model’s ability to regenerate epithelial heterogeneity and lineage differentiation, making it a reliable system for studying cervical epithelial biology.

### scRNA-seq analysis confirms high transcriptional fidelity of patient-derived cervical organoids to native tissue

**Fig. 2.**
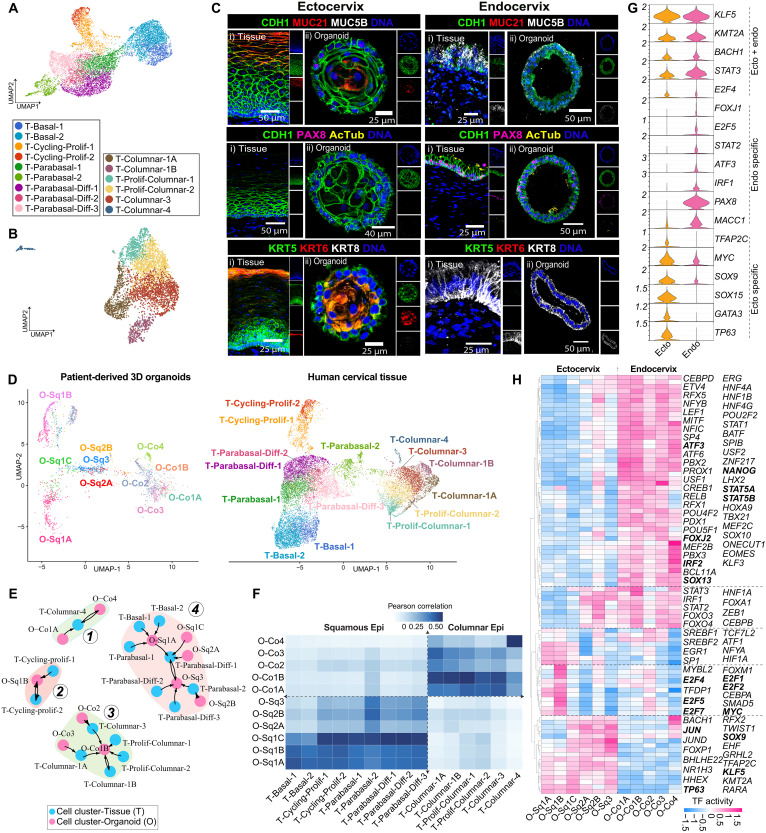
Transcriptional fidelity of patient-derived 3D cervical organoids compared to native cervical tissue. (**A** and **B**) UMAP of tissue-derived squamous (A) and columnar (B) epithelial subclusters; cells colored by cluster annotation. (**C**) IHC images of human ectocervix (left) and endocervix (right) tissues (i) and organoids (ii) stained with CDH1 (green), MUC21(red), MUC5B (gray); and CDH1 (green), PAX8 (magenta), acetylated tubulin (yellow); KRT5 (green), KRT6 (red), KRT8 (gray). Nuclei were stained with 4′,6-diamidino-2-phenylindole (DAPI, blue). (**D**) UMAP projection of IECs from organoids (O-) and tissue (T-) datasets, showing annotated subtypes based on transcriptional identity. (**E**) Directed graph network showing four distinct communities based on transcriptional similarity between organoid-derived (pink) and tissue-derived (blue) epithelial subclusters; each node represents a cell cluster, and arrows indicate the direction of transcriptional similarity. (**F**) Heatmap visualization of Pearson correlation matrix between squamous and columnar epithelial subclusters derived from organoids and tissue. (**G**) Violin plot showing normalized gene expression levels of selected TFs across ecto- and endocervical epithelial subtypes. (**H**) Heatmap depicting the most variable TF activity across squamous and columnar epithelial subclusters; color bar indicates activity levels ranging from high (deep pink) to low (blue).

To further validate the transcriptional similarity between cervical organoids and native tissue, we extracted squamous and columnar epithelial cells from tissue datasets and performed reclustering, identifying nine squamous and six columnar subpopulations (Fig. 2, A and B, and fig. S3, A and B). For clarity and consistency, we uniformly labeled epithelial cells from organoids with “O-” and from tissue with “T-” prefixes (e.g., O-Co, T-Columnar). Among squamous epithelial subtypes, T-Basal-1 and T-Basal-2 strongly expressed basal markers such as *COL17A1*, *TP63*, and *IGFBP6*, and also expressed *JUN*, *SOX4*, and *FOSB*, genes typically associated with parabasal layers, a pattern not observed in basal squamous cells derived from organoids. T-Cycling-Proliferative-1 and -2 displayed high *MKI67* and *CDK1* expression, indicating active proliferation. T-Parabasal-1 and T-Parabasal-2 were characterized by *KRT6A*, and *MMP7* expression, suggesting an intermediate differentiation state. Terminally differentiated squamous cells, T-Parabasal-Diff-1 to -3**,** exhibited high expression of *KRT13*, *SPINK5*, and *CRNN*, highlighting late-stage epithelial maturation (fig. S3C).

Columnar subtypes displayed a distinct transcriptional landscape. T-Columnar-1A expressed *AXIN2* and *PTGS2*, indicating potential epithelial stem/progenitor population (*31*, *32*), while T-Columnar-1B showed strong expression of *MUC5AC*, *MUC5B*, and *AGR2*, indicating mucus-secreting functions. Proliferative populations, T-Proliferative-Columnar-1 and -2, were enriched for *MKI67* and *PCNA*, whereas T-Columnar-3 expressed MHC genes (*CD74* and *HLA-DPB1*), suggestive of antigen-presenting potential. Ciliated cells (T-Columnar-4) expressed *FOXJ1* and *TUBA1A*, confirming their specialized differentiation state (fig. S3D).

To assess the spatial distribution of epithelial subtypes in tissue and organoids, we performed immunohistochemistry (IHC) (Fig. 2C). KRT5 localized to basal and parabasal layers of squamous epithelium, while KRT8 was restricted to columnar cells, consistent with expected lineage identities. KRT6 was enriched in late parabasal and differentiated squamous layers, while MUC21 marked terminally differentiated squamous cells. MUC5B was detected in mucus-secreting columnar cells, while PAX8 and acetylated tubulin confirmed the presence of glandular luminal (*33*–*35*) and ciliated epithelial populations (*36*), respectively, in both organoid and tissue samples.

To assess transcriptional fidelity between epithelial subtypes, we first embedded organoid- and tissue-derived epithelial clusters into a shared Uniform Manifold Approximation and Projection (UMAP) space (Fig. 2D). This revealed substantial overlap, with tight clustering of matched cell types from organoids and tissue, confirming strong transcriptional correspondence. Basal squamous cells from organoids (O-Sq1A) aligned closely with T-Basal-1/2, while proliferative O-Sq1B clustered with T-Cycling-Proliferative-1/2. Parabasal and differentiated squamous populations, including O-Sq1C, O-Sq2A/B, and O-Sq3, mapped to T-Parabasal-1/2 and T-Parabasal-Diff-1/2/3, respectively. Similarly, columnar epithelial subtypes from organoids mapped to their respective tissue counterparts, including O-Co1B with T-Columnar-1B, O-Co2 with T-Columnar-2, and the ciliated O-Co4 population with T-Columnar-4.

To quantify transcriptional similarity between epithelial subtypes of organoid and tissue, we performed cluster similarity analysis by computing similarity scores based on fold-change distributions across shared gene features, ensuring accurate pairing of equivalent cell types across datasets. This analysis revealed four distinct similarity networks, visualized as a community clustering map where nodes represent organoid (O-) or tissue (T-) epithelial populations, and the arrows indicate the direction of transcriptional similarity (Fig. 2E). Cluster 1 connected ciliated epithelial cells O-Co4 and T-Columnar-4, indicating strong similarity. Cluster 2 linked proliferative squamous cells across datasets (O-Sq1B and T-Cycling-Proliferative-1/2), while cluster 3 reinforced the close transcriptional relationship between secretory columnar epithelial cells (O-Co1B and T-Columnar-1B). Cluster 4 grouped basal and parabasal squamous cells, where T-Basal-1/2 and T-Parabasal-1 aligned with O-Sq1A, and O-Sq2B strongly correlated with T-Parabasal-2. In addition, O-Sq3 exhibited strong similarity to T-Parabasal-Diff-3, emphasizing shared features of terminal differentiation. Pearson correlation analysis of differentially expressed genes further confirms high correlation between organoid- and tissue-derived squamous and columnar populations (Fig. 2F).

To investigate lineage-specific transcriptional regulation, we inferred transcription factor (TF) activity from the expression of downstream target genes (Fig. 2, G and H; fig. S3, E and F; and table S4). Clustering based on TF-activities clearly showed separation between ecto- and endocervical epithelial populations, highlighting distinct regulatory programs underlying their identity and maintenance (fig. S3E). *TP63*, *JUN*, *KLF5*, and *SOX15* were predominantly active in squamous epithelia (*37*, *38*), supporting their role in squamous lineage specification. Within squamous subtypes, O-Sq1A and O-Sq1B exhibited high activity of *FOXM1*, *MYC*, and *E2F*, key regulators of cell cycle progression and mitosis (*39*, *40*). In contrast, columnar epithelial subtypes exhibited strong activity of *PAX8*, *FOXJ1*, *HNF4A*, *NANOG*, *STAT5A*, *RFX5*m and *ATF3*, regulators of stem cell fate and ciliogenesis (*41*–*43*). Shared TFs across both lineages, including *TCF7L2*, *ATF1*, *FOXO3*, and *ZEB1*, suggesting common regulatory pathways.

To further characterize regional epithelial identity, we analyzed HOX gene expression patterns, key regulators of development (*44*). HOXB genes were predominantly expressed in columnar epithelium, while HOXA genes were enriched in squamous subtypes (fig. S3, G and H). These patterns reinforce the distinct lineage identities and embryonic origins of ectocervical and endocervical epithelial cells. Together, these findings demonstrate that patient-derived 3D cervical organoids faithfully recapitulate the transcriptional and regulatory landscape of native cervical tissue. By integrating organoid and multiple tissue scRNA-seq datasets, we establish a comprehensive single-cell reference framework of the healthy human cervical epithelium across both ecto- and endocervical regions. This integrative framework offers a valuable benchmark for future studies exploring regional epithelial dynamics, host-pathogen interactions, and disease-associated perturbations.

### Differential innate immune architectures of cervical epithelia inform region-specific mucosal defense

**Fig. 3.**
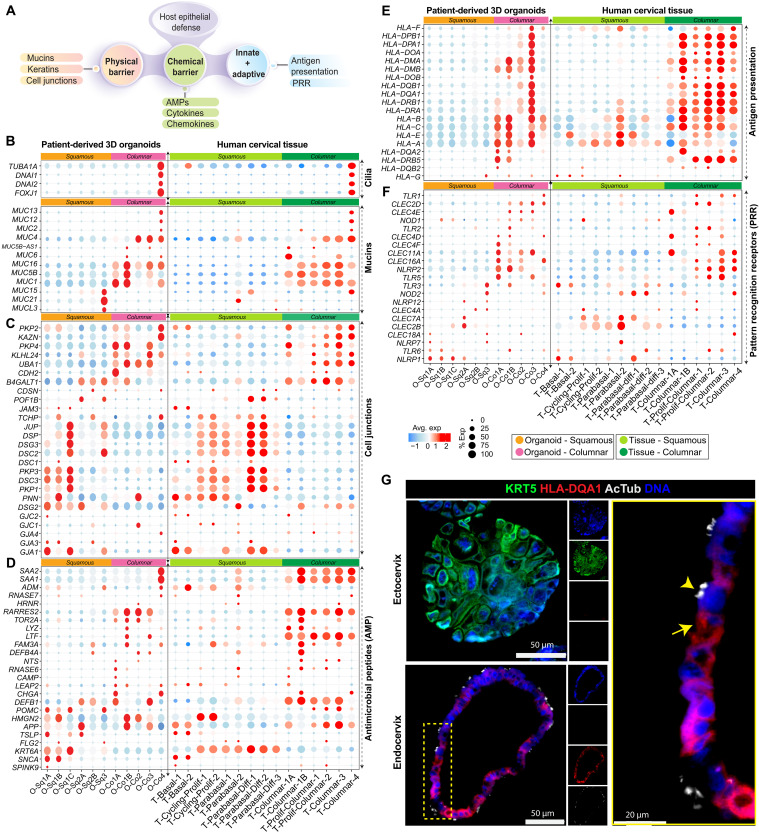
Molecular features of innate immune defense and barrier function in squamous and columnar epithelia of the cervix. (**A**) Schematic highlighting the diverse roles of epithelia in host defense and regulation of innate and adaptive immune response. (**B** to **D**) Dot plot showing the expression levels of markers associated with cilia and mucins (B), cell junctions (C), and AMPs (D) in both squamous and columnar epithelial subpopulations from organoids and tissue datasets; dot size represents the percentage of cells expressing a particular gene; the color bar indicates the intensity of scaled mean expression levels ranging from high (red) to low (blue). (**E** and **F**) Dot plot showing the expression profiles of MHC genes (E) and PRRs (F); circle size indicates the percentage of cells expressing a particular gene, and the color bar represents the intensity of scaled mean expression levels from high (red) to low (blue). (**G**) IHC images of human ectocervical (top left) and endocervical (bottom left) organoids stained for KRT5 (green), HLA-DQA1 (red), and acetylated tubulin (AcTub) (gray); nuclei are counterstained with DAPI (blue). A magnified view of the boxed region from the endocervical organoid is shown (right). Yellow arrow indicates HLA-DQA1^+^/AcTub^−^ epithelial cells, while the yellow arrowhead indicates HLA-DQA1^−^/AcTub^+^ ciliated epithelial cells, indicating distinct epithelial subsets within the organoid model, as identified by scRNA-seq. Images are representative of three biological replicates.

Despite the well-established role of epithelia in host defense, the contribution of ectocervical and endocervical epithelial subtypes to innate immune responses remain poorly defined. To address this, we used microarray and scRNA-seq analysis to profile epithelial innate immune gene expression at both global and single-cell resolution. This approach enabled us to define epithelial subtypes with distinct immune roles, providing a high-resolution view of region-specific immune heterogeneity (Fig. 3, A to F, and fig. S4, A to F).

To assess barrier integrity across squamous and columnar epithelia, we analyzed the expression of mucins, cytokeratins (KRTs), ciliary genes, and intercellular junction markers. Both organoids and tissue exhibited consistent gene expression patterns, validating the reliability of our models. The endocervical columnar epithelium expressed higher levels of both gel-forming and membrane-bound mucins such as *MUC1*, *MUC4*, *MUC5B*, *MUC6*, *MUC12*, *MUC13*, and *MUC16*, compared to the ectocervical cells (*45*). Notably, *MUC2*, *MUC12*, and *MUC13* were enriched in O-Co4 and T-Columnar-4 cells, while *MUCL3*, *MUC15*, and *MUC21* were predominantly expressed in differentiated squamous cells (O-Sq3 and T-Parabasal-diff-3) (Fig. 3B and fig. S4A). KRTs, critical for mechanical stability and epithelial integrity (*46*), exhibited distinct expression patterns across ectocervical and endocervical subtypes (fig. S4B). Analysis of cell-cell junctions, including gap, tight, and adherens junctions, revealed distinct organization between ectocervical and endocervical epithelia (Fig. 3C and fig. S4C). In the ectocervix, *GJA1*, *GJA3*, *DSG2*, *DSC1*–*3*, *CLDN5*, and *CLDN8* were enriched in basal and parabasal layers, whereas differentiated squamous cells exhibited reduced expression. In contrast, endocervical columnar cells showed robust expression of *GJC1*, *CDH2*, *UBA1*, *PKP4*, and *KAZN*, reflecting a unique structural organization of these cervical epithelia.

A key component of innate host defense is the secretion of antimicrobial peptides (AMPs), which bridge innate and adaptive immunity (*47*). Our analysis of AMP profiles between squamous and columnar epithelial subsets revealed further divergence in immune capacity (Fig. 3D and fig. S4D). Endocervical columnar cells exhibited strong expression of *DEFB1*, *DEFB4A*, *CHGA*, *LTF*, *LYZ*, *TOR2A*, *HRNR*, and *RARRES2*, suggesting a highly active antimicrobial defense (*48*). Ciliated cells uniquely expressed *SAA1* and *SAA2*, reinforcing their specialized immune surveillance role. Conversely, ectocervical squamous cells displayed limited AMP expression, with *SNCA*, *TSLP*, and *SPINK9* detected, suggesting a greater reliance on physical barrier integrity rather than antimicrobial secretion.

Growing evidence suggests that epithelial cells contribute to antigen presentation, influencing both CD8^+^ and CD4^+^ T cell activation (*24*, *49*). Our analysis revealed that endocervical columnar cells exhibit higher MHC expression compared to ectocervical squamous cells (Fig. 3E and fig. S4, E and F). Quantitative reverse transcription polymerase chain reaction (qRT-PCR) validation confirmed strong MHC expression in endocervical cells, with scRNA-seq identifying O-Co3 cells as np-APCs, exhibiting up-regulated MHC class I and II genes (fig. S4G). These observations were further supported by IHC, which revealed spatially restricted HLA-DQA1 expression enriched in a specific subset of endocervical epithelial cells, likely corresponding to Co3, and consistently higher in endocervical organoids compared to ectocervical organoids (Fig. 3G). This pattern corroborates findings from scRNA-seq, microarray, and qRT-PCR analyses. In contrast, ciliated epithelial cells (Co4) showed markedly lower HLA-DQA1 expression, reinforcing the transcriptional and protein-level distinction between columnar subsets. This highlights a previously underappreciated immunomodulatory role of the endocervical epithelium in antigen presentation and mucosal immune priming.

To further characterize pathogen recognition, we examined the expression of pattern recognition receptors (PRRs), including Toll-like receptors (TLRs), nucleotide-binding oligomerization domain–like receptors (NLRs), and retinoic acid–inducible gene-I–like receptors (RLRs) (Fig. 3F). *TLR1* and *TLR2* expression was notably higher in columnar epithelial cells, a pattern observed across qRT-PCR, microarray, and scRNA-seq data (Fig. 3F and fig. S5, A and B). Additional PRRs including *TLR5*, *NLRP2*, *CLEC16A*, and *CLEC4F*, were expressed in columnar subtypes (O-Co1A to O-Co3), reinforcing their role in bacterial recognition and immune activation. These findings align with known ligand specificities, including *TLR2* sensing Gram-positive bacterial components (*50*) and *TLR5*, which has been implicated in sensing flagellated microbes such as *Mobiluncus* species, known to colonize the female genital tract and modulate innate immune responses (*51*–*53*). Notably, ciliated cells (O-Co4) expressed *TLR1*, *CLEC2D*, and *CLEC4E*, suggesting further immune specialization. In contrast, ectocervical basal squamous cells (O-Sq1A-1C) exhibited high *NLRP1* and *TLR6* expression, while parabasal and differentiated squamous cells expressed *TLR3*, *NOD2*, *CLEC2B*, and *CLEC7A*, indicating distinct PRR repertoires across cervical epithelial subtypes (Fig. 3F).

We next analyzed cytokine and chemokine expression across tissue and organoid (fig. S5C), revealing distinct inflammatory and immunomodulatory signatures across ecto- and endocervix. In ectocervical basal squamous cells (O-Sq1A-C), genes linked to inflammatory regulation, including *CCL26*, *IL18*, *IL1RAP*, *IL20RB*, and *IL31RA*, were highly expressed (fig. S5C) (*54*). Parabasal and differentiated squamous cells exhibited high *CXCL14*, *CXCL6*, *CXCL17*, *CXCR2*, *IL1B*, and IL36RN expression. In contrast, endocervical epithelial cells showed strong expression of *CXCL1*, *CXCL3*, *CXCL8*, and *CCL28*, with O-Co4 cells displaying the highest expression. In addition, O-Co1A cells exhibited *CCL2*, *IL23A*, *IL10*, *IL1R1*, *IL1RL2*, *IL18R1*, and *IL1A*, reinforcing their role in immune modulation. *CCL2* expression was consistently higher in endocervical compared to ectocervical samples, as validated across qRT-PCR and microarray datasets (fig. S5, D and F). While cytokine and chemokine expression were largely consistent between tissues and organoids, certain genes displayed subcluster-specific differences, emphasizing the influential role of tissue microenvironmental cues on epithelial immune regulation.

Together, these findings demonstrate regional specialization of innate immune profiles within the ectocervical and endocervical epithelia, highlighting differences in barrier formation, AMP secretion, antigen presentation, and cytokine signaling. These compartmentalized responses likely contribute to the protection of the FRT against infections.

### Decoding cell type–specific responses of ecto- and endocervical epithelia to Chlamydia infection

**Fig. 4.**
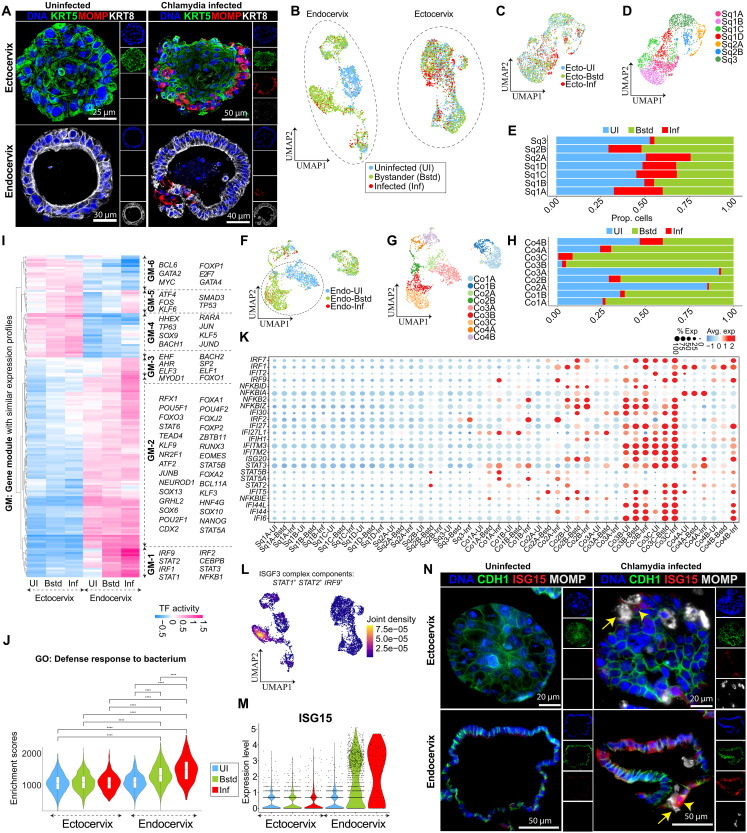
Single-cell transcriptomics reveals epithelial subtype–specific responses to Chlamydia infection. (**A**) Immunofluorescence images of ectocervical (top) and endocervical (bottom) organoids, uninfected (left) or infected (right) for 48 hours with Chlamydia, stained for KRT5 (green), major outer membrane protein (MOMP) (red), KRT8 (gray), and DAPI (blue). (**B**) UMAP projection of single cells from ecto- and endocervical organoids, colored by infection status: uninfected (UI), infected (Inf), and bystander (Bstd). (**C** and **D**) UMAP showing reclustered ectocervical squamous epithelial population from (B), colored by infection status (C) and subtype identity (D). (**E**) Proportion of UI, Bstd, and Inf cells in each ectocervical squamous subtype. (**F** to **G**) UMAP showing reclustered endocervical columnar epithelia from (B), colored by infection status (F) and subtype (G). (**H**) Proportion of UI, Bstd, and Inf cells in each endocervical columnar subtype. (**I**) Heatmap of differentially regulated TFs between ecto- and endocervix across infection conditions; color bar depicts the TF activity scores from high (deep pink) to low (blue). (**J**) Violin plot of gene set enrichment scores for the GO term defense response to bacterium across epithelial compartments and infection states; statistical significance assessed by Wilcoxon rank-sum test with Holm-adjusted *P* values (****P ≤ 0.0001). (**K**) The relative expression of IFN-related genes across ecto- and endocervical subclusters; dot size represents the % of cells expressing a particular gene, and the color bar indicates the intensity of scaled mean expression levels ranging from high (red) to low (blue). (**L**) Gene-weighted density UMAP projections showing expression of *STAT1*, *STAT2*, and *IRF9* across epithelial cells in (B). (**M**) Violin plot showing ISG15 expression across ecto- and endocervical organoids in uninfected, bystander, and infected states. (**N**) IHC images showing CDH1 (green), ISG15 (red), MOMP (gray), and DAPI (blue) in ecto- and endocervical organoids, uninfected (left) or infected (right). Yellow arrows mark infected cells; arrowheads indicate ISG15^+^ bystander cells.

To investigate epithelial subtype–specific responses to infections, we evaluated Chlamydia-infected patient–derived ecto- and endocervical organoids (Fig. 4A and fig. S6A). In ectocervical organoids, Chlamydial inclusions initially appeared in basal layers at 24 hours postinfection (hpi), progressively spreading to adjacent layers by 48 hpi and causing structural disorganization by 4 to 5 days postinfection (dpi). In contrast, endocervical organoids exhibited increasing GFP fluorescence intensity, indicating intracellular bacterial replication, followed by luminal extrusion of inclusions. Notably, endocervical epithelial cells showed lower infection rates and maintained structural integrity (fig. S6A), suggesting intrinsic differences in susceptibility and response between the two cervical epithelial types.

scRNA-seq analysis revealed compartment-specific transcriptional responses to Chlamydia infection (Fig. 4, B to H, and fig. S6, B and C). Within the ectocervical epithelium, seven transcriptionally distinct squamous subclusters (Sq1A-D, Sq2A-B, and Sq3) were identified (Fig. 4, C and D, and fig. S6, D and E). Notably, Chlamydia infected all squamous subtypes, and pseudotime analysis confirmed that differentiation trajectories remained largely unaltered following infection (Fig. 4E and fig. S6D). In contrast, endocervical epithelia exhibited pronounced transcriptional remodeling, particularly in uninfected bystander cells, which clustered separately from infected cells in UMAP projections (Fig. 4F). Nine distinct columnar subpopulations (Co1A-B, Co2A-B, Co3A-C, Co4A-B) were identified (Fig. 4G and fig. S6, F and G), with Co3B and Co3C emerging as transcriptionally remodeled bystander subsets exhibiting unique gene expression signatures (fig. S6G). Pseudotime analysis confirmed that while overall lineage differentiation was preserved, Co3 subsets underwent infection-induced molecular reprogramming. Infections were predominantly localized to the ciliated Co4A and Co4B subpopulations, whereas non-ciliated subsets exhibited relatively low infection rates (Fig. 4H). Differential gene expression analysis further delineated infection-induced transcriptional signatures across all epithelial subsets (fig. S6, H and I, and tables S5 and S6).

To identify key transcriptional regulators underlying epithelial subtype–specific infection responses, we performed TF activity analysis, revealing five major gene modules (GMs) that distinguished ecto- and endocervical epithelial responses (Fig. 4I and fig. S6J). GM-1 was characterized by a robust IFN response, with enriched activity of *IRF1*, *IRF2*, *IRF9*, *NFKB1*, *STAT1*, and *STAT2* in both infected and bystander endocervical cells. This pattern suggests paracrine IFN signaling, wherein infected cells induce IFN-stimulated genes (ISGs) in neighboring epithelial subsets via type I and type III IFN pathways, a known mechanism of epithelial defense (*55*, *56*). GM-2 and GM-3 were predominantly associated with columnar epithelial responses and included regulators such as *ATF2*, *CDX2*, *FOXJ2*, and *AHR*, with infection-induced up-regulation of TFs such as *SPI1*, *ESRRA*, *RFX1*, and *RARA*. In contrast, GM-4 and GM-5 were primarily associated with squamous epithelial responses, featuring TFs such as *GATA2*, *GATA4*, *SOX9*, and *TP63*, essential for stratified epithelial maintenance. Notably, GM-5 included stress-responsive TFs such as *KLF4*, *ATF4*, *SMAD3*, *FOS*, and *TP53*, which exhibited increased activity across both ectocervical and endocervical subtypes postinfection (Fig. 4I and table S7).

To further delineate biological pathways activated in response to Chlamydia infection, we performed enrichment analysis, revealing a clear divergence between squamous and columnar epithelial responses. The enrichment for the GO term “defense response to bacterium” was statistically significant in infected and bystander endocervical populations (Fig. 4J). In particular, Co3B and Co3C subsets exhibited strong activation of immune pathways, including IFN-α and -γ responses, interleukin-6 (IL-6)–Janus kinase–signal transducers and activators of transcription (STAT) signaling, inflammatory response, and tumor necrosis factor–α (TNF-α) signaling via nuclear factor κB (fig. S7A). In contrast, squamous epithelial populations showed enrichment for Notch and transforming growth factor–β (TGF-β) signaling, pathways essential for epithelial stratification and barrier homeostasis (fig. S7A and table S8) (*4*). Proliferative subsets, such as Co2A and Sq1C-Sq1D, showed enrichment for E2F target genes and G_2_-M checkpoint pathways, highlighting an active proliferative response to infection (fig. S7A). Conversely, ciliated endocervical subsets (Co4A–B) showed down-regulation of cilium organization and motility-related pathways postinfection, as confirmed by both microarray and scRNA-seq analyses (fig. S7, B and C).

At the transcriptional level, columnar subsets, particularly Co3B, Co3C, and infected Co4B, displayed strong up-regulation of ISGs, including *IFI6*, *IFI27*, *IFITM2*, *IFITM3*, *ISG20*, and *IFI30* (Fig. 4K and fig. S7D). Notably, uninfected bystander cells exhibited comparable ISG expression levels to infected cells, further supporting the hypothesis that paracrine IFN signaling propagates antimicrobial responses throughout the epithelium. In contrast, squamous epithelia showed minimal ISG activation and maintained high *TP63* activity regardless of infection status. Sq1D, Sq2A, and Sq3 subsets exhibited elevated *TFAP2C* activity, while Sq1A-D subsets demonstrated increased activity of *MYC* and *SP1* (fig. S7D and table S9). Columnar subsets exhibited strong up-regulation of TFs such as *IRF9*, *STAT2*, *IRF1*, *IRF2*, and *NFKB1*, with the highest activity observed in remodeled populations. Co3B and Co3C cells strongly expressed *STAT1*, *STAT2*, and *IRF9* (Fig. 4L), suggesting potential assembly of the ISGF3 complex, which translocates to the nucleus to activate ISG transcription. We further validated the ISG marker ISG15, which was strongly expressed in endocervical bystander and infected epithelial cells compared to the ectocervix (Fig. 4M and fig. S7E). IHC further confirmed robust ISG15 induction in noninfected bystander cells adjacent to infected regions in both ecto- and endocervical organoids, with particularly strong widespread activation across endocervical epithelial cells and only mild induction in the ectocervix (Fig. 4N). This spatial pattern supports paracrine IFN signaling as a driver of bystander activation, priming the epithelium for coordinated antimicrobial defense. Together, these findings highlight epithelial subtype–specific, IFN-driven immune responses to Chlamydia infection, which may help restrict pathogen spread and reinforce mucosal immunity.

These findings reveal notable epithelial subtype–specific immune responses to Chlamydia infection. Notably, bystander columnar cells undergo extensive transcriptional reprogramming, mounting a robust proinflammatory and IFN-driven response that may serve to limit infection spread and reinforce mucosal immunity. Overall, the transcriptional immune response to Chlamydia infection is notably stronger in endocervical epithelial subsets than in their squamous counterparts of the ectocervix.

### Innate immune and antimicrobial responses of cervical epithelial subtypes to Chlamydia infection

**Fig. 5.**
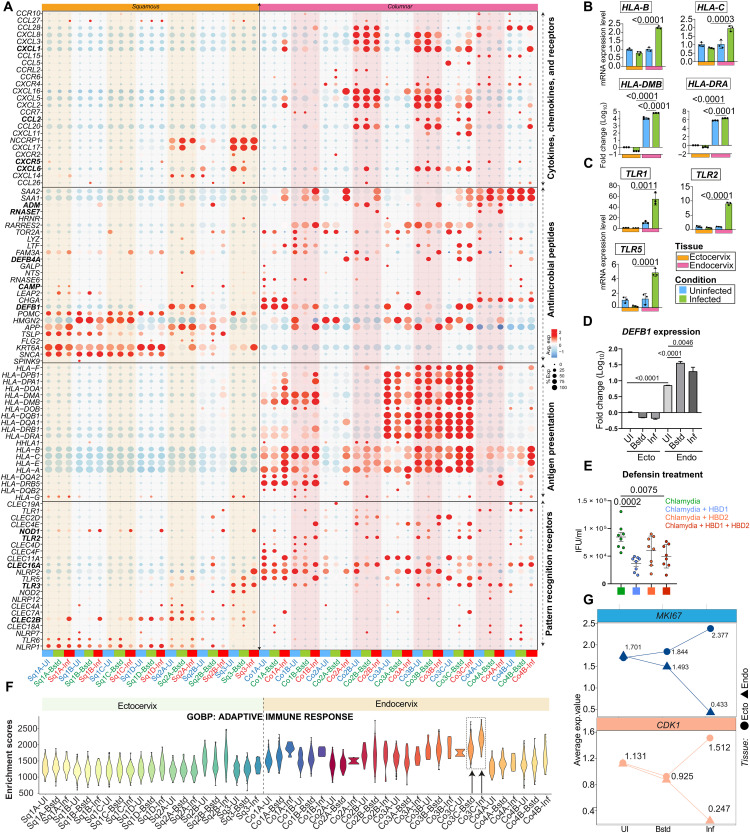
Chlamydia infection alters innate immune and proliferative programs across cervical epithelial subtypes. (**A**) Dot plot showing mRNA profiles of PRRs, MHC genes, AMPs, and cytokines across ecto- and endocervical epithelial subtypes and UI/Bstd/Inf conditions. The size of each dot reflects the percentage of cells expressing a specific gene, and the color bar signifies the intensity of scaled mean expression levels, ranging from high (red) to low (blue). Cluster labels are colored by infection status: blue for uninfected (UI), green for bystander (Bstd), and red for infected (Inf). (**B** and **C**) qRT-PCR analysis of MHC genes (*HLA-B*, *HLA-C*, *HLA-DMB*, and *HLA-DRA*) (B); PRRs (*TLR1*, *TLR2*, and *TLR5*) (C) expression in uninfected and infected ecto- and endocervical organoids at 48 hpi. (**D**) qRT-PCR analysis of *DEFB1* expression in FACS-sorted uninfected, infected, and bystander epithelial cells after 36 hpi. Data represent means ± SD from three technical replicates normalized to ectocervix control, and *P* values were calculated using Student’s *t* test. (**E**) Infectivity assay of Chlamydia lysates from ectocervical organoids, with or without pretreatment with HBDs (HBD1, HBD2, or both) at 5 dpi. Data represent means ± SD from eight nonoverlapping regions across two replicates. (**F**) Violin plot showing gene set enrichment scores for the GO term related to the adaptive immune response across epithelial subclusters under different infection conditions. (**G**) Trend plot showing changes in mean expression levels of selected proliferation markers *MKI67* and *CDK1* under different infection conditions; lines are colored by gene; point shapes distinguish ectocervical (circles) and endocervical (triangles) samples.

To investigate the impact of Chlamydia infection on innate immune responses across epithelial subpopulations, we analyzed gene expression profiles of defense-related markers. Our findings revealed distinct, cell type–specific immune responses, with both infected and bystander cells showing strong up-regulation of innate immunity-associated genes. Notably, epithelial subtypes that expressed mucins and tight junction markers under homeostatic conditions retained their expression postinfection but at elevated levels in both infected and bystander populations (Fig. 5A and fig. S8A). For example, differentiated squamous epithelial cells (Sq3) maintained expression of mucins such as *MUC15* and *MUC21*, with increased expression relative to uninfected controls. Similarly, basal and parabasal squamous cells (Sq1 and Sq2) exhibited a pronounced up-regulation of PRR genes, including *NLRP1* and *TLR6*. In contrast, endocervical columnar cells exhibited increased expression of *TLR2*, *TLR5*, and *CLEC4F*, with up-regulation observed in both infected and bystander subsets, indicating an active role in bacterial recognition and immune signaling. MHC class I and II gene expression were strongly up-regulated in Co1A-B and Co3A-C subpopulations, suggesting enhanced antigen-presenting capacity in columnar epithelial cells, potentially mediated by bystander cell activation (Fig. 5A). Consistent with these findings, qRT-PCR confirmed the strong induction of MHC markers and PRR genes in endocervical columnar epithelia, aligning with both microarray and scRNA-seq data (Fig. 5, B and C, and fig. S8, B and C).

Further analysis of chemical barrier components, including AMPs, cytokines, and chemokines revealed their up-regulation across subsets of both infected and bystander cells in the ectocervix and endocervix. (Fig. 5A and fig. S8, A to E). Specifically, in the ectocervix, genes such as *CLEC2B* were highly expressed in infected cells of Sq1A-C, *NOD1* in bystander and infected cells of Sq1B-D, Sq2B/3, *CAMP* in infected Sq1A/2A-B cells, *ADM* in infected cells of Sq1B and bystander cells of Sq2A-B, and *CXCR5* in bystander Sq2B/3 cells. In the endocervix, we observed up-regulation of *TLR2* in bystander cells of Co2B/3C, *TLR3* (Co3C/4A), *CLEC16A* (bystander Co3B-C), *DEFB1* (Co1B/3C), *DEFB4A* (Co2A/3B-C), *RNASE7* (infected Co2B/4A), *CXCL6* (bystander Co2B; bystander and infected Co3B-C), *CCL2* (bystander and infected Co3C), *CXCL1* (Co2A/3C), and *IL1B* (bystander and infected Co2B/3B-C). Among these, human β-defensin 1 (HBD1) gene *DEFB1*, an AMP crucial for innate immune response showed particularly strong expression in bystander cells of specific endocervical subsets (Co3C) (Fig. 5, A and D). To assess the functional impact of increased defensin expression, Chlamydia was treated with HBD1 and HBD2 before infection. Chlamydia harvested from cells infected with defensin-treated bacteria exhibited reduced infectivity compared to untreated controls (Fig. 5E), indicating that defensins secreted by endocervical epithelial cells can effectively limit Chlamydia infectivity and potentially prevent reinfection.

Pathway enrichment analysis revealed stronger activation of adaptive immune response in endocervical epithelia compared to ectocervical cells, with the highest response observed in Co3C bystander and infected populations (Fig. 5F). Among squamous subtypes, Sq2B exhibited the highest immune response scores, suggesting a potential role in coordinating epithelial-immune interactions.

Chlamydia infection has previously been associated with epithelial hyperproliferation (*15*). In line with this, we observed increased expression of proliferation-related markers in infected ectocervical squamous epithelia. However, infected endocervical columnar cells displayed reduced expression of proliferative genes (Fig. 5G and fig. S8F). These findings suggest that Chlamydia may differentially regulate epithelial proliferation in a cell type–specific manner, possibly influencing tissue remodeling and long-term infection outcomes.

Together, these results highlight the distinct, cell type–specific innate immune responses of ectocervical and endocervical epithelial subtypes following Chlamydia infection. While squamous epithelial cells prioritize barrier integrity and PRR signaling, columnar cells exhibit a more immune-competent phenotype characterized by enhanced antigen presentation and defensin-mediated antimicrobial defense. These findings underscore the role of epithelial subpopulations in shaping host-pathogen interactions and suggest that the differential immune responses of cervical epithelial compartments may influence infection outcomes and susceptibility to reinfection.

### Coordinated intercellular communication among epithelial subtypes enhances immune and tissue regenerative signaling

Epithelial cell-cell interactions are fundamental for maintaining tissue integrity and coordinating immune responses during infections. Toward this, we used CellChat (*57*) to analyze intercellular signaling networks across homeostatic and Chlamydia-infected states in ecto- and endocervical epithelial subpopulations. We observed a substantial increase in the number of signaling interactions postinfection in both epithelial compartments, suggesting dynamic remodeling of intercellular communication (fig. S9, A and B). To dissect these interactions, epithelial subpopulations were categorized as signal senders (cells expressing ligands initiating signaling) and signal receivers (cells expressing cognate receptors that activate downstream pathways). In the ectocervix, signaling pathways such as FGF, CD46, JAM, and DES were consistently enriched under both conditions, whereas IL-10, AGRN, and EPGN were exclusive to uninfected states and absent postinfection (fig. S9C). In contrast, pathways such as SEMA4, SEMA6, CADM, and EPHA emerged postinfection, indicating Chlamydia-driven rewiring of epithelial communication (fig. S9, C and E, and table S10). Among squamous subtypes, basal Sq1A cells actively engaged in both outgoing and incoming signals involving LAMININ, epidermal growth factor (EGF), and migration inhibition factor (MIF), with signaling strength remaining relatively unchanged after infection (fig. S9E). However, NOTCH signaling exhibited an infection-specific shift, with basal Sq1A cells as primarily signal senders, targeting Sq1C and Sq3 terminally differentiated populations, supporting its role in epithelial stratification (*4*).

Endocervical columnar epithelia displayed distinct infection-induced signaling adaptations. Pathways such as IL-1, TNF, NCAM, TRAIL, and GALECTIN were strongly enriched during infection, whereas SEMA4, CDH5, DES, and TGF-β were predominant in uninfected conditions (fig. S9, D and F, and table S11). Notably, Co3 cells emerged as key regulators of IL-1, TNF, OCLN, and GALECTIN signaling (fig. S9F). IL-1 signaling, specifically initiated by Co3 cells, targeted Co1A stem cells, suggesting a potential role in both immune activation and regenerative responses.

**Fig. 6.**
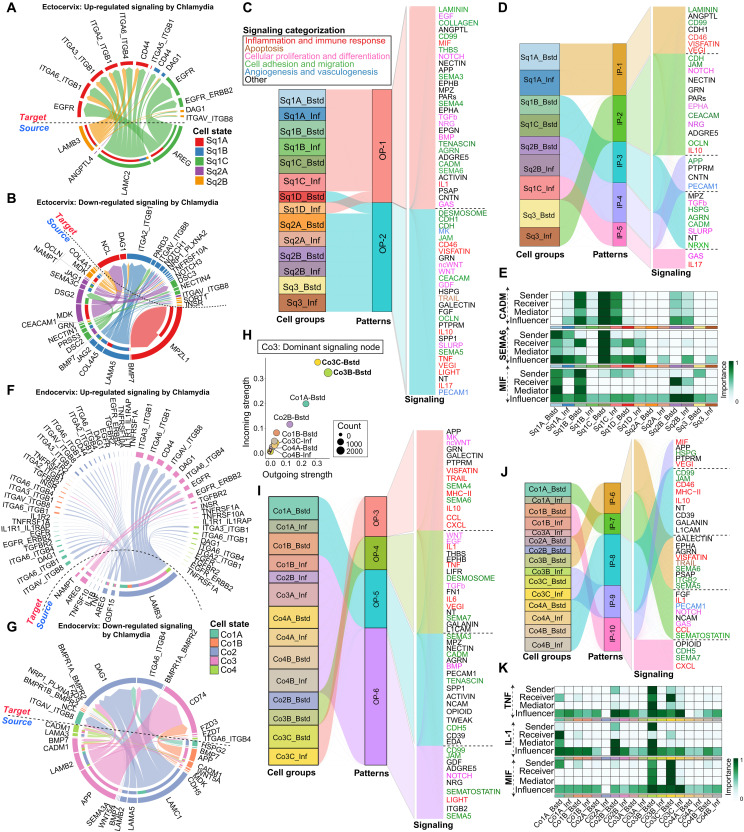
Cell-cell communication analysis reveals differential signaling patterns and networks among ecto- and endocervical epithelial subtypes during Chlamydia infection. (**A** and **B**) Chord diagrams showing up-regulated (A) and down-regulated (B) L-R signaling pairs in ectocervical squamous epithelial cells upon Chlamydia infection; outer bars indicate signal sending (ligand-expressing) cell groups; inner bars colored by receiving (receptor-expressing) cell groups; edges colored by signaling source/senders. (**C** and **D**) River plots depicting the inferred outgoing (C) and incoming (D) communication patterns in ectocervical squamous epithelia, highlighting the associations between latent patterns, cell groups, and enriched signaling pathways; thickness of the flow reflects the contribution of cell groups or signaling pathways to each pattern, with pathway labels colored by signaling category. (**E**) Heatmap showing the relative importance and signaling role of each cell group in the MIF, SEMA6, and CADM signaling networks in the ectocervix; scale bar color denotes the criticality of a cell group in driving the communication network ranging from high (dark green) to low (gray). (**F** and **G**) Chord diagrams illustrating up-regulated (F) and down-regulated (G) L-R signaling interactions across endocervical columnar epithelial subpopulations after infection. (**H**) Scatter plot visualization of predominant signal senders and receivers among endocervical epithelial subsets in 2D space; circle size represents the total inferred links (outgoing and incoming) associated with each cell type, colored by subclusters. (**I** and **J**) Inferred outgoing (I) and incoming (J) communication patterns in endocervical epithelia, illustrating the links between latent patterns, cell types, and signaling pathways. (**K**) Heatmap visualization of the relative importance and signaling role of each cell group in the endocervix for IL-1, MIF, and TNF signaling networks. Scale bar color as in (E).

To further dissect epithelial communication networks, we examined L-R interactions in squamous and columnar epithelia before and after Chlamydia infection. In squamous epithelial cells, we identified 17 up-regulated and 30 down-regulated L-R pairs postinfection. Basal (Sq1C) and parabasal (Sq2A-B) cells up-regulated ligands such as *AREG*, *LAMB3*, *LAMC2*, and *ANGPTL4*, which are associated with tissue repair and epithelial integrity (Fig. 6A) (*58*). Conversely, basal cells exhibited down-regulation of antibacterial and differentiation-associated ligands, including *MPZL1*, *BMP7*, *COL4A5*, *DSC2*, and *JAG2* (Fig. 6B) (*59*, *60*). Similarly, parabasal cells exhibited down-regulated levels of *DSG2*, *JAG1*, *OCLN*, and *COL4A1*. While direct evidence in Chlamydia infection is limited, DSG2 and OCLN are known to regulate epithelial tight junction integrity, and their down-regulation may compromise barrier function and increase susceptibility to infection (*61*–*64*).

Pattern recognition–based signaling analysis in ectocervical epithelia identified two outgoing (OP-1 and OP-2) and five incoming (IP-1 to IP-5) signaling patterns (Fig. 6, C and D). In OP-1, basal Sq1A-C cells were key signal senders, regardless of infection status, actively secreting bone morphogenetic protein (BMP), TGF-β, SEMA3, and MIF, which regulate immune responses and epithelial regeneration (Fig. 6C) (*65*, *66*). OP-2, predominantly driven by parabasal and terminally differentiated cells, mediated immune and barrier-associated pathways, including CD46, IL-17, and JAM. The incoming signaling patterns (IP-1 to IP-5) further emphasized the coordinated response among squamous epithelial subtypes during infection (Fig. 6D). For example, IP-1 was specific to basal Sq1A cells, mediating self-coordination via CD99, CD46, and CDH1 signaling. IP-2, targeting terminally differentiated Sq3 cells, involved JAM and NOTCH, reinforcing epithelial barrier integrity, while IP-3 engaged bystander Sq1B and Sq1C cells via TGF-β, AGRN, and SLURP signaling, implicated in tissue remodeling and immune modulation (*65*, *67*). IP-4, associated with PECAM1, was prominent in parabasal Sq2B cells, while IP-5 was unique to infected, highly proliferative Sq1C cells, targeted by IL-17–mediated signaling.

To investigate how infected epithelial cells influence neighboring bystander populations, we mapped directional L-R interactions originating from infected cells and targeting bystander cells (fig. S9G). TNF signaling from Sq2B-infected cells targeted bystander cells expressing TNFRSF1A, while Sq1A and Sq3-infected cells triggered the CD46-JAG1 axis, influencing Sq1A-D bystander cells. The strongest infection-induced interaction involved MIF signaling, where infected epithelial cells expressed MIF, which engage CD74/CD44 receptors on basal (Sq1A-B) and parabasal (Sq2B) bystanders. Network centrality analysis highlighted the critical role of bystander epithelial cells in mediating immune and tissue repair responses, particularly via MIF, SEMA6, and CADM signaling (Fig. 6E and fig. S9G) (*68*, *69*).

In endocervical columnar epithelia, we identified 15 up-regulated and 19 down-regulated L-R pairs postinfection. *LAMB3*, *AREG*, *TNF*, and *IL1B* were primarily up-regulated in Co2 and Co3 subpopulations, indicating their role as key signal senders (Fig. 6F). Conversely, *HSPG2*, which is known to facilitate pathogen entry (*70*, *71*), was down-regulated in Co1A stem-like cells. Laminin-associated ligands (*LAMC1*, *LAMA5*, and *LAMB2*) and *CADM1*, both implicated in pathogen binding and susceptibility (*72*, *73*), were also reduced across Co2-Co4 populations (Fig. 6G). Remodeled bystander cells of Co3B-3C emerged as dominant signal sending and receiving population, mediating immune and tissue regenerative signaling (Fig. 6H and fig. S9F). However, their functional roles in coordinating infection responses require further elucidation.

We further identified four outgoing (OP-3 to OP-6) and five incoming (IP-6 to IP-10) signaling patterns in columnar epithelia (Fig. 6, I and J). Unlike squamous epithelial signaling, columnar epithelial communication was strongly influenced by infection status. Remodeled Co3C cells emerged as key signal senders in OP-3, driving immune-related pathways including IL-10, CCL, CXCL, MHC-II, TRAIL, and SEMA4. OP-4 was exclusive to bystander Co2B and Co3B cells, mediating IL-1, TNF, TGF-β, and EGF signaling (Fig. 6I). Among the incoming signals, IP-6 involved bystander Co2A-B and Co3B populations responding to SEMA5, SEMA6, and GALECTIN signals, while IP-7 linked Co1A stem-like cells to IL-1 and CCL-mediated inflammatory signaling (Fig. 6J). Notably, nearly all infected cell types signaled to Co3B and Co3C bystander populations through MIF-(CD74^+^CD44), a pathway critical for mucosal healing and wound repair (Fig. 6K and fig. S9H) (*74*). In parallel, TNF signaling from infected Co2B, Co3B, and Co3C cells activated neighboring bystander cells (fig. S9H).

Our study reveals distinct intercellular signaling networks within squamous and columnar epithelia during both homeostasis and Chlamydia infection. While both epithelial lineages engage in signaling pathways that support barrier integrity and tissue repair, endocervical columnar epithelia exhibit a more dynamic and infection-responsive communication profile. Notably, transcriptionally remodeled Co3B and Co3C bystander cells exhibited a unique coordination mechanism, activating a broader set of inflammatory and immune response pathways than their squamous counterparts.

## DISCUSSION

**Fig. 7.**
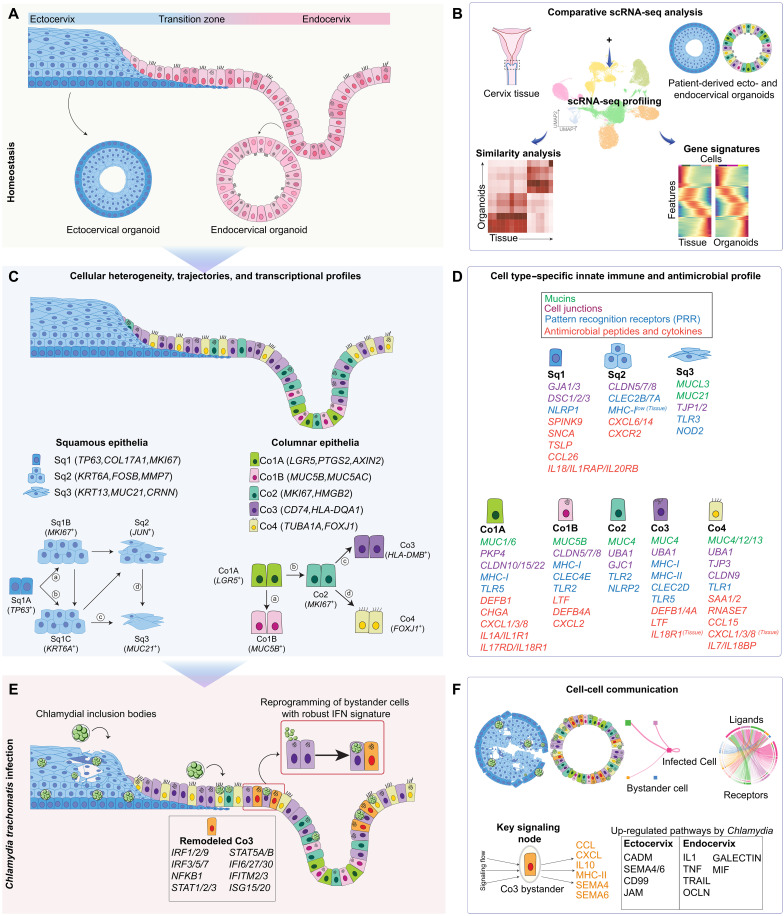
Graphical summary of cervical epithelial cellular diversity, innate mucosal immune features, and Chlamydia-induced responses. (**A**) Anatomical overview of the human uterine cervix highlighting the ectocervix and endocervix, their distinct epithelial compositions, and convergence at the transition zone. Patient-derived 3D organoids derived from adult epithelial stem cells preserve region-specific epithelial architecture and identity. (**B**) Integration of organoid and cervical tissue scRNA-seq datasets reveals high transcriptional fidelity and molecular congruence across corresponding epithelial subsets. Gene signature and similarity analyses demonstrate that organoids recapitulate both cellular heterogeneity and native epithelial programs. (**C**) Depiction shows the identified lineage-specific epithelial subtypes across squamous and columnar compartments under homeostasis. Pseudotime trajectory analysis reveals hierarchical differentiation from basal/stem-like cells to terminally differentiated states, with key subtype-defining transcriptional markers shown. (**D**) Depiction highlighting region- and cell type–resolved expression profiles of innate immune defense genes at steady state. Distinct epithelial subtypes show selective enrichment of mucins, junctional components, PRRs, AMPs, and cytokines, reflecting compartmentalized mucosal immune strategies. (**E**) Schematic showing that Chlamydia infection remodels the epithelial landscape. In the endocervix, transcriptional reprogramming of uninfected bystander cells gives rise to IFN-responsive columnar subsets with robust ISG expression, hallmarks of paracrine immune activation*.* (**F**) Infection reconfigures epithelial communication networks. Cell-cell interaction modeling reveals that columnar bystander cells function as major signaling contributors mediating immune and regenerative responses through pathways such as CCL, CXCL, MHC-II, MIF, SEMA, TNF, and IL-1. Region-specific shifts in L-R interactions underscore divergent mucosal responses between ecto- and endocervix.

By integrating patient-derived 3D cervical organoids with single-cell transcriptomics and native tissue analysis, our study provides an in-depth, region-resolved high-resolution atlas of cervical epithelial heterogeneity and infection-induced immune dynamics. We show that organoid-derived squamous and columnar epithelial subtypes faithfully recapitulate the molecular, transcriptional, and spatial features of native tissue, supporting the use of these models for interrogating anatomical region–specific host-pathogen interactions. Our findings reveal that stratified ectocervical and columnar endocervical epithelia adopt distinct intrinsic immune strategies to limit infection, preserve tissue integrity, and coordinate responses across cellular compartments (Fig. 7).

Our findings demonstrate that cervical epithelial heterogeneity arises not only from lineage-specific differentiation trajectories but also from regionally distinct immunological specializations. Ectocervical squamous epithelia prioritize and maintain a structural defense strategy enriched in barrier-associated genes and signaling pathways such as NOTCH and TGF-β (*4*). In contrast, endocervical columnar cells displayed pronounced immune competence, characterized by strong baseline and infection-induced expression of mucins, PRRs, cytokines, AMPs, and MHC genes. These findings suggest that endocervical columnar epithelia not only provide physical and chemical defense but also contribute to antigen presentation and immune priming at mucosal surfaces (*49*, *75*).

Infection with Chlamydia revealed substantial differences in epithelial responses between cervical compartments. In ectocervical organoids, infection was broadly distributed but did not alter differentiation trajectories. The response was characterized by up-regulation of stress-responsive TFs (e.g., *TP53*, *KLF4*, and *ATF4*) and innate sensors (e.g., *NLRP1* and *TLR6*) in specific squamous subsets such as Sq1A, Sq1C-D, and a modest increase in PRR and AMP expression while maintaining stratification signatures. This subdued response could reflect a compartmentalized defense strategy optimized for preserving tissue architecture while controlling infection. One of the most notable findings of this study is the extensive transcriptional remodeling observed in endocervical bystander cells following infection. Despite being uninfected, Co3B and Co3C cells gained a distinct immune-activated state enriched for ISGs, defensins, proinflammatory cytokines, and MHC molecules, and exhibited activation of key IFN signaling TFs such as *STAT1*, *STAT2*, and *IRF9*, components of the ISGF3 complex. These features indicate a robust paracrine activation of bystander cells, likely driven by type I and type III IFNs from infected neighbors, as has been observed in other epithelial systems (*76*–*78*). Bystander cells often mounted immune responses comparable to or stronger than infected cells (e.g., ISG15 expression), emphasizing their critical role in amplifying mucosal defense.

This phenomenon of epithelial reprogramming was further reflected in the cell-cell interaction landscape. Chlamydia infection extensively reshaped the L-R networks, with selective induction and suppression of signaling pathways. In the squamous epithelium of ectocervix, infection promoted CADM, SEMA4, SEMA6, CD99, and JAM signaling, indicative of epithelial remodeling and barrier restoration (*68*, *79*). NOTCH signaling with a known role in promoting epithelial differentiation and stratification in cervix and esophagus tissues (*4*, *18*) was activated along the basal-to-suprabasal axis postinfection, suggesting a repair-oriented program in response to infection-induced epithelial injury. In endocervical epithelia, transcriptionally remodeled Co3B and Co3C bystander subsets emerged as central regulators of infection response. These cells received MIF, TNF, and GAS6 signals from infected neighbors and, in turn, initiated immunomodulatory signaling via IL-10, CXCL, and MHC-II pathways. In parallel, activation of the GAS6-MERTK axis in Co1A stem-like bystander cells points to a regenerative feedback loop regulating inflammation and promoting epithelial repair (*80*). Functionally, we show that epithelial immune gene activation translates to antimicrobial activity. β-Defensin (*DEFB1*) expression was robustly induced in endocervical organoids and its exogenous application significantly reduced Chlamydia infectivity when tested in vitro. Given that defensins can also recruit dendritic and T cells (*81*), these findings suggest a dual role in direct pathogen restriction and immune cell recruitment, bridging innate and adaptive immunity.

Together, our findings establish that cervical epithelial subtypes are not passive barriers but are active sensors and regulators of mucosal immunity. They engage in cell-intrinsic defenses, transmit paracrine signals to modulate bystander activation, and coordinate tissue-level communication to limit infection and support regeneration. The compartmentalization of these responses reflects adaptation to the distinct anatomical and functional roles of the ecto- and endocervix. From a translational perspective, these findings offer a framework for identifying immune-competent epithelial subsets (e.g., Co3B/C and Sq2B) and key signaling nodes (e.g., Co3C and Sq1A), as potential targets for modulating mucosal immunity. IFN signaling and MIF-CD74/CD44 interactions pathways may be leveraged for therapeutic intervention. Moreover, the epithelial remodeling signatures uncovered here may serve as biomarkers of infection-induced dysregulation, with implications for predicting persistent infection, metaplasia, or disease progression to conditions like cancer.

Thus, our study establishes a scalable, region-specific cervical organoid platform integrated with single-cell transcriptomics to dissect host-pathogen interactions. Future extensions incorporating immune cells, microbiota, controlled hormonal environments, or coinfections (e.g., HPV) will enable more comprehensive modeling of epithelial-immune cross-talk, mucosal homeostasis and infection susceptibility. Collectively, our findings reveal a highly coordinated, epithelial-intrinsic immune architecture in the human cervix that governs defense and regeneration during infection, informing next-generation mucosal vaccines and epithelial-targeted interventions to protect reproductive health and prevent infection-associated disorders.

## MATERIALS AND METHODS

### Antibodies and chemicals

The following primary antibodies were used for immunofluorescence: mouse anti–acetylated tubulin–Alexa Fluor 647 (1:300, Santa Cruz Biotechnology, sc-23950-AF647), mouse anti–E-cadherin–Alexa Fluor 488 (1:50, BD Biosciences, 560061), mouse anti–E-cadherin (1:50, BD Biosciences, 610181), rabbit anti–KRT5–Alexa Fluor 488 (1:300, Abcam, ab193894), mouse anti-MUC5B (1:200, Abcam, ab77995), rabbit anti-MUC21 (1:200, ProteinAtlas, HPA052028), rabbit anti-KRT8 (1:200, Abcam, ab59400), mouse-anti-KRT6 (1:50, Abcam, ab18586), recombinant rabbit anti-PAX8 (1:200, Abcam, ab239363), goat anti–*C. trachomatis* major outer membrane protein (1:500, Bio-Rad, 1990-0804), rabbit anti–HLA-DQA1 antibody (EPR7300) (1:200, Abcam, ab128959), rabbit anti-ISG15 polyclonal antibody (1:200, Proteintech,15981-1-AP), and for labeling the DNA, 4′,6-diamidino-2-phenylindole (DAPI, Roche, 10236276001) were used. The following secondary antibodies were used for immunofluorescence: donkey anti-goat Cy3 (1:150, Dianova, 705-165-003), donkey anti-mouse Cy3 (1:150, Dianova, 715-166-020), donkey anti-mouse Alexa Fluor 647 (1:150, Dianova, 715-605-150), donkey anti-rabbit Alexa Fluor 647 (1:150, Dianova, 711-605-152), goat anti-mouse Cy3 (1:150, Dianova, 115-165-006), goat anti-rabbit Cy3 (1:150, Dianova, 111-165-144), goat anti-rabbit Alexa Fluor 647 (1:150, Dianova, 111-606-045), and donkey anti-rabbit Cy3 (1:150, Dianova, 711-165-152).

### Immunofluorescence histochemistry

Immunofluorescence staining was performed on paraffin-embedded tissue and organoid sections as described previously (*14*, *15*). Images were acquired with Leica TCS SP5 confocal microscope and processed with ImageJ.

### qRT-PCR analysis

Total RNA of the uninfected and Chlamydia-infected human ectocervical and endocervical organoid pellets or flow cytometric sorted cell pellets were isolated using AllPrep DNA/RNA Mini Kit (QIAGEN, #80204) according to the manufacturer’s protocol. Using the RevertAid First-Strand cDNA Synthesis Kit (Fisher Scientific, K1621), 1 μg of uninfected and infected human ecto- and endocervix RNA was converted into cDNA according to the manufacturer’s protocol. qRT-PCR was performed using the following reaction mixture: 12.5 ng of cDNA, 3.6 μl of respective forward and reverse primer mix (10 μM) mix, 10 μl of GreenMasterMix (2×) High ROX for qPCR (Genexxon Biosciences, M3052.0000), and made up the volume to 20 μl of DNase/RNase-free water. The following qRT-PCR cycle program was used: 95°C for 10 min, followed by 40 cycles at 95°C for 15 s and 60°C for 60 s. The melting cycle of 95°C for 15 s, 60°C for 16 s, and 95°C for 15 s were used. The expression level of human GAPDH (glyceraldehyde 3-phosphate dehydrogenase), a housekeeping gene, was used to normalize the relative mRNA levels of respective genes in the uninfected and Chlamydia-infected human ecto- and endocervix organoids. Following primers for GAPDH were used as controls: forward 5′-GGTATCGTGGAAGGACTCATGAC-3′, reverse: 5′-ATGCCAGTGAGCTTCCCGTTCAG-3′; All samples were measured as triplicates. The forward and reverse primers were designed with a melting temperature of 60°C from Sigma-Aldrich (100 μM) and were diluted to 10 μM before use. The following primer pairs were used: TLR1- forward: 5′-AGTTGTCAGCGATGTGTTCG-3′, reverse: 5′-AAAATCCAAATGCAGGAACG-3′, TLR2- forward: 5′-GGTTCAAGCCCCTTTCTTCT-3′, reverse: 5′-TTCCCACTCTCAGGATTTGC-3′, reverse 5′- TTTCCAGAGCCGTGCTAAGT-3′, TLR5- forward: 5′- TGCCACTGTTGAGTGCAAGTC-3′, reverse: 5′-ACCTGGAGAAGCCGAAGGTAAG-3′, HLA-B- forward: 5′-ATACCTGGAGAACGGGAAGG-3′, reverse: 5′-CAGTGTCCTGAGTTTGGTCCT-3′, HLA-C- forward: 5′-ATTACATCGCCCTGAACGAG-3′, reverse: 5′-GTGTGTCTTTGGGGGTTCTG-3′, HLA-DMB- forward: 5′-CTCACAGCACCTCAACCAAA-3′, reverse: 5′- AGAAGCCCCACACATAGCAG-3′, HLA-DRA- forward: 5′-GAAATGGAAAACCTGTCACCA-3′, reverse: 5′-GCATCAAACTCCCAGTGCTT-3′, CCL2- forward: 5′-CCCCAGTCACCTGCTGTTAT-3′, reverse: GAGTTTGGGTTTGCTTGTCC-3′, DEFB1- forward: 5′- AGATGGCCTCAGGTGGTAAC-3′, reverse: 5′-CACTTGGCCTTCCCTCTGTA-3′. Relative mRNA levels of the samples were measured in terms of fold-change [2^-(∆∆Ct)] by SYBR-Green detection with qRT-PCR, StepOnePlus Real-Time PCR System (Applied BiosystemsTM), and StepOneTM Software (v2.3, Applied Biosystems).

### Culturing of organoids and *C. trachomatis* infection

Human cervical tissue samples were provided by the Department of Gynecology, Charité University Hospital, and August-Viktoria Klinikum, Berlin. Usage for scientific research was approved by their ethics committee (EA1/059/15), and informed consent was obtained from all subjects. The study complies with all relevant ethical regulations regarding research involving human participants. Biopsies were sourced from standard surgical procedures. Only anatomically normal tissue biopsies from anonymous female donors aged between 35 and 75 years undergoing gynecological procedures for benign conditions were processed within 2 to 3 hours after removal. In line with ethical approvals and anonymization protocols, detailed clinical metadata such as menopausal status or surgical indication were not available to investigators. Organoids were generated from ecto- and endocervical biopsies, processed and independently obtained from a total of six individuals. While some ecto- and endocervical tissues may have originated from the same donor, samples were not patient-matched, as the study focused on region-specific epithelial responses. The ecto- and endocervix organoids were cultured according to the previously described methods (*14*). For the infection with the *C. trachomatis*–L2-GFP strain, organoids were harvested, and Matrigel was removed. For the human endocervix, organoids were broken down before infection by passing the organoid suspension through a 26G needle four times. Organoids were infected at multiplicity of infection (MOI) of 5 in an infection medium [Advanced Dulbecco’s modified Eagle’s medium/F12 + Hepes (12 mM) + Glutamax (1×) + 5% heat-inactivated fetal calf serum] for 2 hours at 37°C on a shaker (~100 rpm). Following infection, the organoids were washed, seeded on Matrigel, and allowed to grow in a 3D media for 36 hours for scRNA-seq and FACS or up to 5 days for bulk transcriptomic analysis and paraffinization.

### AMP treatment and infectivity assay

For AMP treatment, the required amount of bacteria was pretreated with either HBD1 (25 μg/ml, Peptanova, #4337-s) in the presence of 2 mM dithiothreitol or HBD2 (25 μg/ml, Peptanova, #4338-s) or in the presence of both in SPG buffer for 30 min at room temperature. Human endocervical organoids were infected with pretreated bacteria at MOI of 5 for 2 hours in the infection medium. Organoids were washed and allowed to grow in Matrigel for 48 hours. After 48 hpi, organoids were harvested and centrifuged into pellet. Pellet was transferred into 15-ml falcon tubes with 1 ml of infection medium. Approximately 1 ml of sterile glass beads were added to flacon tubes and vortexed for 4 min. Cell suspension was diluted to 1:2. Fifty microliters of diluted sample was added to previously grown HeLa cells monolayers in 96-well plates and incubated for 2 hours. The media were replaced with complete media and incubated for 24 hours. After 24 hpi, the media were removed, added 200 μl of ice-cold methanol incubated plate at 4°C overnight. Methanol was washed twice with 200 μl of 1× phosphate-buffered saline (PBS). The cells were stained with DAPI (1:1000) in 0.2% bovine serum albumin (BSA) solution in PBS for 1 hours. The cells were washed with 1× PBS, images were taken from the fluorescent microscope, and infectivity per milliliter was calculated.

### Flow cytometric sorting of *C. trachomatis*–infected cells

Organoids were incubated with 2 ml of TrypLE in a shaker (20 min, 37°C, 180 rpm for ecto cervix and 10 min, 37°C, 180 rpm for endo cervix organoids). The organoids were made into single cells by pipetting up and down 20 times using a 1-ml pipette tip and diluted with 5 ml of 0.04% BSA-PBS solution. After inverting, the cells were passed through a 40-μm cell strainer. The suspension was transferred to 75-mm Falcon tubes with cell strainer caps (Fisher Scientific) and placed on ice. Cells were sorted for GFP-positive and -negative cells into a Falcon tube containing ice-cold 0.04% BSA-PBS solution using BD FACSAria III Cell Sorter (BDbiosciences) running the FACSDiva software (BDbiosciences). Using side-scatter and forward-scatter properties, any cell debris, doublets, or aggregates were excluded. Cells were sorted using GFP (502LP-530/30) filters for positive and negative cells.

### Microarray expression profiling and analysis

Organoids were washed, pelleted, and resuspended in 1-ml TRIzol reagent (Invitrogen, #15596026). RNA was isolated using the Allprep and RNeasy Mini Kit (Qiagen, #80204) according to the manufacturer’s protocol, and quality was assessed by Agilent 2100 Bioanalyzer with RNA Nano 6000 microfluidics kit (Agilent Technologies, #5067-1511). Microarray experiments were performed as previously described (*15*). Single-color hybridizations were conducted using custom 60K Agilent SurePrint G3 v2 arrays. Raw intensity data were subjected to background correction, quantile normalization, and differential gene expression analysis (with *P* value < 0.05 and 1.5-fold change) of the data were performed using the R package LIMMA (*82*). Overrepresentation analysis was performed using the function “compareCluster” from the R package ClusterProfiler (*83*) using default settings with significant cutoffs for *P* value and adjusted *P* value (Benjamin-Hochberg–based correction) < 0.05.

### scRNA-seq sample preparation

Organoids from three biological replicates of human ectocervical cells and two biological replicates of human endocervical cells were harvested (0.04% BSA-PBS) after Chlamydia infection. Single cells were prepared from the organoids as above in 0.04% BSA-PBS solution. After inverting, the cells were passed through a 40-μm cell strainer. Single cells were pooled at an equal ratio from biological samples and processed for the Cell Multiplexing Oligo (CMO) labeling using kit 3′ CellPlex Kit Set A (10x Genomics, #PN-1000261). CMO-labeled cells were pooled at an equal ratio, made the cell concentration to 2000 cell/μl, and proceeded for the library preparation. Single cells were partitioned into nanoliter-scale Gel-Bead-In-EMulsions using a 10X Chromium Controller. The library was prepared using Single-Cell 3′ reagent kit v3 for reverse transcription, cDNA amplification, and library construction according to the manufacturer’s protocol. Sequencing was performed in paired-end mode with an S1 100-cycles kit using Illumina Novaseq 6000 sequencer.

### Bioinformatic analysis of scRNA-seq data from patient-derived cervical organoids

#### Processing of raw sequencing data and downstream analysis

The CellRanger (v.6.1.1) software from the 10X Genomics platform was used to demultiplex and process the raw sequencing data of uninfected and Chlamydia-infected ecto- and endocervical organoid samples. We used the “cellranger multi” pipeline with default parameters. Reads were aligned to the 10X reference human genome build GRCh38-2020-A.

Downstream analysis for the generated digital gene expression matrices was performed using the R package Seurat (v.4.1.1) (*84*). Primarily, we scrutinized for potential doublets by neglecting barcodes with fewer than 100 genes, more than 8000 genes, and more than 80,000 Unique Molecular Identifier (UMI) counts. Cells were filtered out if their UMIs derived from the mitochondrial genome exceeded 20%. In addition, we assessed and excluded the erroneously annotated barcodes, i.e., cells/doublets with a substantial and coherent expression of a hybrid transcriptome based on columnar and squamous epithelial markers such as *LGR5*/*KRT8*/*18* and *TP63*/*KRT5*/*14*, respectively. As an outcome, 4985 cells were used for further analyses.

Normalization and variance stabilization of the count data were performed using the R package sctransform (v.0.3.3) (*85*), which also identified the highly variable genes. We regressed out the mitochondrial mapping percentage and cell cycle scores during data normalization and scaling. Dimensionality reduction, clustering, and data visualization were performed using the “RunPCA,” “FindNeighbors,” “FindClusters,” and “RunUMAP” functions on the top 30 principal components, with default settings. Last, by repeating the same workflow, we reclustered ecto- and endocervical epithelial cell groups separately to identify the subpopulations present during steady state and infection.

#### Differential expression analysis and GSEA

Differentially expressed genes between cell types and clusters were identified using the “FindAllMarkers” function from the Seurat R package, with default parameters. GSEA was performed on scRNA-seq count data using the default functions provided by the R package escape (v.1.4.1) (*86*). The joint gene-weighted density estimation of multiple features was visualized using the R package Nebulosa (v.1.2.0) (*87*).

#### Signature scoring analysis

Gene signature scoring for our scRNA-seq data was performed using the R package Ucell (v.1.1.1) (*88*) based on the Mann-Whitney *U* statistic. Since the scoring technique is rank based, the signature scores are independent of the cellular heterogeneity in the data as they are interpreted as relative expressions of the gene set within an individual cell’s transcriptome. We applied this scoring method to identify the uninfected bystander cells present within the Chlamydia-infected samples, which usually contain both the infected and uninfected bystander cells. Using the purely uninfected and FACS-sorted infected cells, we collected the uninfected and infected cell-specific gene signatures for each cell cluster via differential gene expression analysis. Next, we used the “AddModuleScore_UCell” function to perform gene set scoring in Chlamydia-infected samples. Infected cells had scores higher than defined cutoffs (ranging from the minimum value to the first quartile), whereas the bystander cells secured lower scores and failed to surpass the cutoff value. Later, this information was added back to the scRNA-seq metadata, and the cells from Chlamydia-infected samples were tagged as either bystander or infected cells. Further, we also collected hallmark gene sets of “E2F_targets” and “G2M_checkpoint” for enrichment analysis.

#### Cell-cell interaction analysis

We used CellChat’s (v.1.1.3) (*57*) standard pipeline to decode cell-cell communication between squamous and columnar epithelial subsets of uninfected and infected organoids. First, we created individual CellChat objects and preprocessed their expression matrix using the built-in functions “identifyOverExpressedGenes,” “identifyOverExpressedInteractions,” and “projectData” with default settings to identify potential interactions. We calculated communication probabilities to infer the active signaling networks using the functions “computeCommunProb,” “computeCommunProbPathway,” and “aggregateNet.” In addition, we removed the effect of cell proportion by setting the parameter “population.size” to true during probability calculation. We used the “netAnalysis_signallingRole” function to identify the potential source and targets involved in signaling. Using the “identifyCommunicationPatterns” function, we deduced the coordination between cell types and signaling pathways. Last, we applied a comparative CellChat framework to comprehensively understand the signaling changes across cell groups pre- and postinfection. Next, we used the function identifyOverExpressedGenes to perform differential expression analysis, which revealed the up- and down-regulated L-R pairs between the two biological conditions (i.e., uninfected and infected).

#### Cell transition trajectory and pseudotime analysis

Developmental trajectories in our data were modeled using the R package URD (v.1.1.1) (*89*). We used the R package destiny (v. 3.9.1) (*90*) to calculate the transition probabilities between cells and construct the diffusion map. The function “plotDimArray” was used to visualize the diffusion components. Next, we performed pseudotime analysis using the standard built-in functions by defining a set of cells as the starting point (root) and possible end point (tip) based on the expression of stem cell and highly differentiated cell type markers, respectively. Specifically, for the ectocervical organoids, we used the squamous lineage specific stem-cell marker, *TP63*^+^ basal epithelial cells (cluster O-Sq1A) as the root, while for endocervical organoids, we used *LGR5*^+^ cells (cluster O-Co1A). The possible or potential trajectory end points (tips) were defined using terminally differentiated subtypes based on marker expression, specifically, *KRT13*/*MUC21*^+^ cells (O-Sq3) for the squamous lineage and *TUBA1A*^+^ ciliated cells (O-Co4) for the columnar lineage. URD tree structure was generated and visualized using the “buildTree” and “plotTree” functions. Eventually, we modeled the temporal gene regulation using the “plotSmoothFit” function.

#### TF activity analysis

TF activities in ecto- and endocervical epithelia were investigated using the R package Dorothea (v. 1.4.2) (*91*). Initially, we collected regulons from DoRothEA human regulon database with the highest confidence levels A to C for each TF-target interaction. Here, TF activity is a proxy of the transcriptional state of its direct targets. We calculated the viper scores on Dorothea regulons using the wrapper function “run_viper.” Next, we identified the highly variable TFs across cell clusters by calculating the variance of average TF activity scores across subclusters. The top 30 most variable TFs per cluster were selected for downstream visualization. These values were then transformed into *z* scores for better visualization of results using a heatmap.

#### Integrative analysis with cervical tissue scRNA-seq data

To assess transcriptional similarities between in vivo cervical tissue and 3D organoid models, we used publicly available scRNA-seq cervical tissue datasets from five sources (*26*–*30*). Only healthy cervical tissue samples were included for data integration and analysis. For integration with our organoid scRNA-seq data, we used Seurat v5 (*92*) to address potential batch effects. Each dataset, including our organoid data, was initially processed independently following Seurat’s standard log-normalization workflow. The datasets were subsequently merged into a single Seurat object using the “merge” function, with distinct layers assigned to each dataset via the “split” function. Independent normalization and variable feature detection were then performed across the datasets.

We then performed integrative analysis using the ‘IntegrateLayers’ function with ‘Harmony-based’ integration method. Clustering was performed using the FindNeighbors and FindClusters functions based on the top 15 principal components, with a resolution of 0.1. Nonlinear dimensionality reduction was performed using the RunUMAP function for visualization. As a result, a total of 109,594 integrated cells were obtained. Since the external datasets contained additional cell types such as stromal, immune, endothelial, and smooth muscle cells, we first extracted epithelial cell clusters by identifying epithelial marker genes (*KRT5*, *KRT14*, and *KRT13* for squamous epithelia; *KRT7*, *KRT8*, *KRT18*, and *PAX8* for columnar epithelia). Reclustering of epithelial cells from both the organoid and external tissue data was performed using the same integration and clustering workflow. Differential expression analysis was performed by rejoining layers within the Seurat object using the “JoinLayers” function, allowing for subsequent downstream analyses. Cluster similarity between organoid and tissue subpopulations was quantified using the R package ClusterFoldSimilarity with default parameters (*93*). Cell type annotations for identified clusters were performed on the basis of canonical marker gene expression or coexpression, supplemented by information from the Human Protein Atlas database. Further, the clustered gene expression plots were generated using the R package scCustomize (*94*).

### Statistical analysis and reproducibility

GraphPad Prism (v.8) was used for statistical calculations and the generation of plots. The data are displayed as means ± SEM. *P* < 0.05 was considered statistically significant.
