# Supplementary material for: Single-cell atlas of cervical organoids uncovers epithelial immune heterogeneity and intercellular cross-talk during Chlamydia infection
Source: Sci Adv. 2025 Oct 3;11(40):eady1640. doi: 10.1126/sciadv.ady1640 (PMC12494012; doi:10.1126/sciadv.ady1640)
Supplement: Supplementary file 1 — Figs. S1 to S9 Legends for tables S1 to S11 Legend for data S1 [file sciadv.ady1640_sm.pdf]

Science Advances
AAAS

# Supplementary Materials for

## Single-cell atlas of cervical organoids uncovers epithelial immune heterogeneity and intercellular cross-talk during Chlamydia infection

Pon Ganish Prakash *et al.*

Corresponding author: Cindrilla Chumduri, cindrilla.chumduri@bce.au.dk

*Sci. Adv.* **11**, eady1640 (2025)
DOI: 10.1126/sciadv.ady1640

**The PDF file includes:**

Figs. S1 to S9
Legends for tables S1 to S11
Legend for data S1

**Other Supplementary Material for this manuscript includes the following:**

Tables S1 to S11
Data S1

## Figure S1

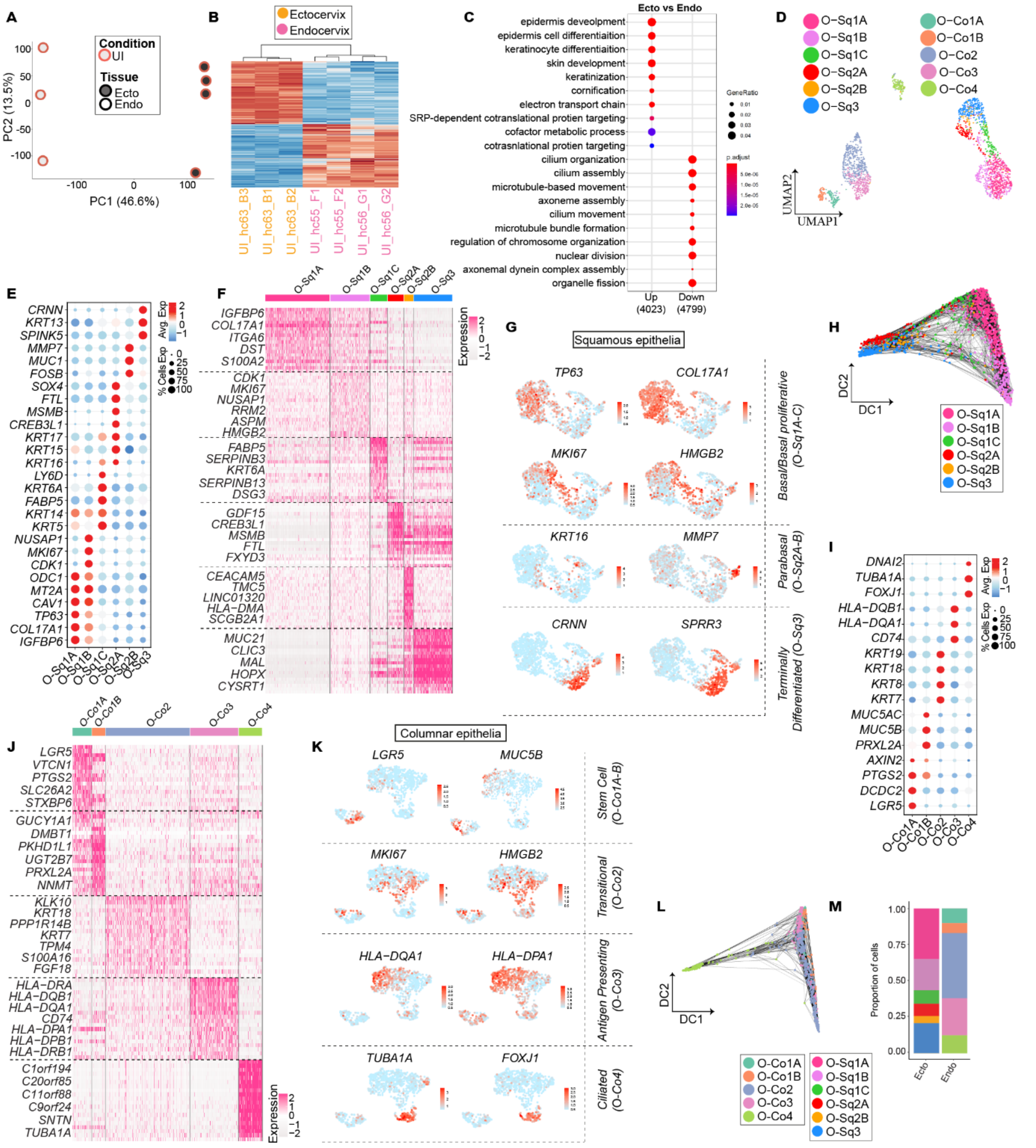


**Figure S1: Characterization of transcriptional landscapes and epithelial subtype heterogeneity in the ecto- and endocervix.**

**A)** Principal component analysis (PCA) of microarray data reveals global transcriptional differences between ecto- and endocervical organoids. **B)** Heatmap of differentially expressed genes (DEGs) between ecto- and endocervical organoids; columns represent biological replicates. **C)** Dot plot showing enriched gene ontology (GO) terms for biological processes

associated with ecto- and endocervix; dot size indicates gene ratio and fill color denotes adjusted P-value. **D)** UMAP projection of all epithelial subclusters from ecto- and endocervix; each dot represents a single cell and is colored by cluster identity. **E)** Dot plot showing the relative expression of canonical markers for squamous epithelial subtypes; circle size represents the percentage of cells expressing a particular gene, the color bar indicates the intensity of scaled mean expression levels ranging from high (red) to low (blue). **F)** Heatmap highlighting the top 5 DEGs between ectocervical epithelial subclusters; columns represent single cells grouped as clusters, and the color bar denotes the expression range from high (pink) to low (grey). **G)** Feature plot showing normalized expression of squamous cell subtype-specific markers in ectocervix; the color bar represents gene expression levels ranging from low (blue) to high (red). **H)** Diffusion map (DM) illustrates the branching differentiation trajectories of squamous epithelial cells of ectocervical organoids. **I)** Dot plot depicting the relative expression of endocervical columnar epithelial subtype markers. **J)** Expression heatmap showing the top 5 DEGs between endocervical epithelial subtypes; color scale as in (F). **K)** Feature plot showing normalized expression values of columnar epithelial cell subtype-specific markers in endocervix; color scale as in (G). **L)** DM depicting the differentiation trajectories of columnar epithelial cells of endocervical organoids. **M)** Bar graph showing the proportional distribution of epithelial subtypes within ecto- and endocervical organoid populations.

7
7

## Figure S2

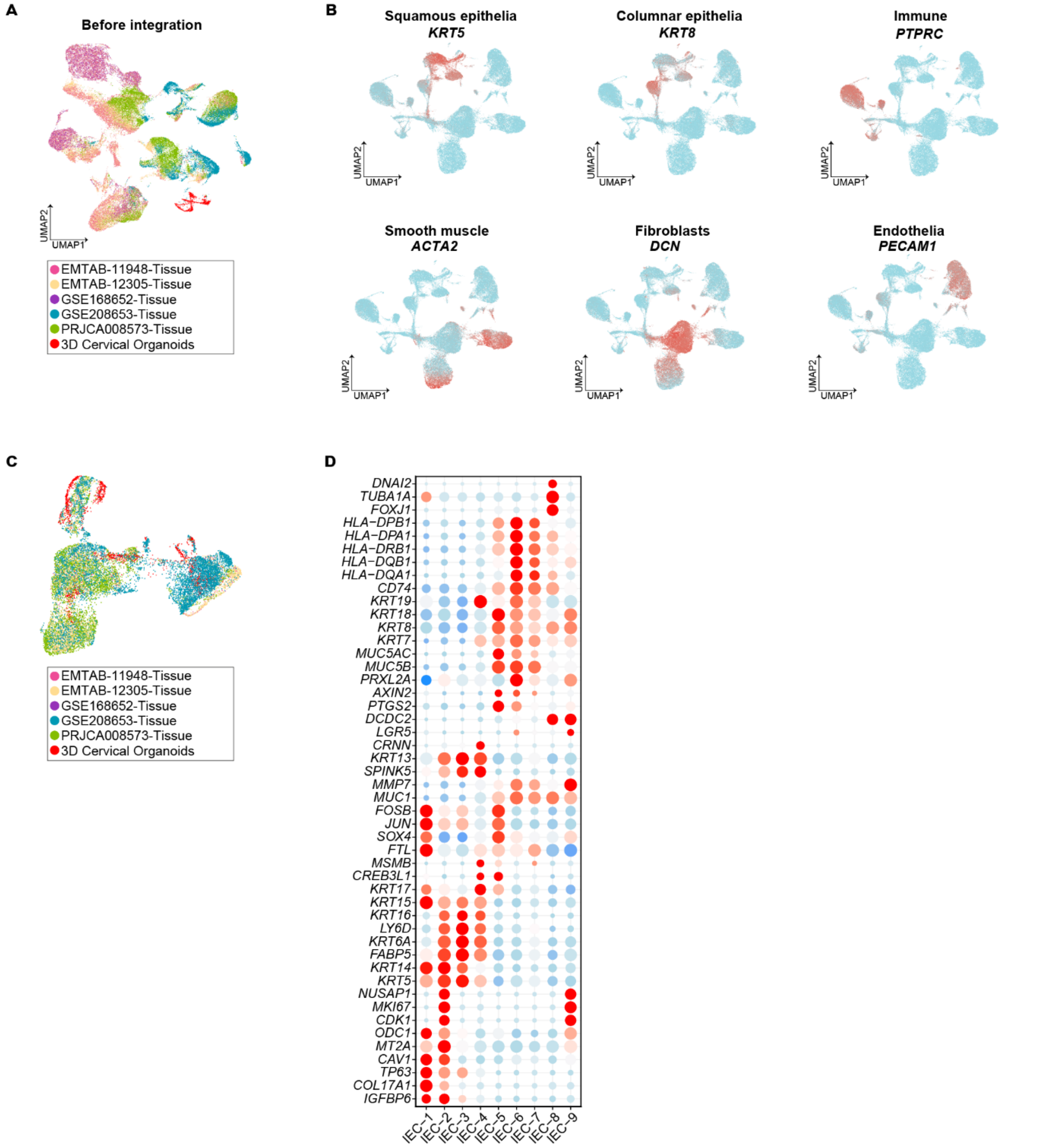


**Figure S2: Integration of human cervical tissue and organoid scRNA-seq datasets reveals shared epithelial transcriptional signatures.**

**A)** UMAP projection of cervical epithelial cells from five human tissue datasets and organoids before integration, illustrating batch-driven clustering and poor cross-dataset alignment. **B)** Feature plots depicting normalized expression of canonical markers for epithelial cells (*KRT5/KRT8*), immune cells (*PTPRC*), smooth muscle cells (*ACTA2*), stromal (*DCN*) and endothelial cells (*PECAM1*). Color scale indicates expression levels from low (blue) to high

(red). **C)** UMAP projection of integrated epithelial subclusters, with cells colored by their dataset of origin. **D)** Dot plot showing the relative expression of canonical epithelial markers used for cluster annotation; circle size represents the percentage of cells expressing a particular gene, the color bar indicates the intensity of scaled mean expression levels ranging from high (red) to low (blue).

## Figure S3

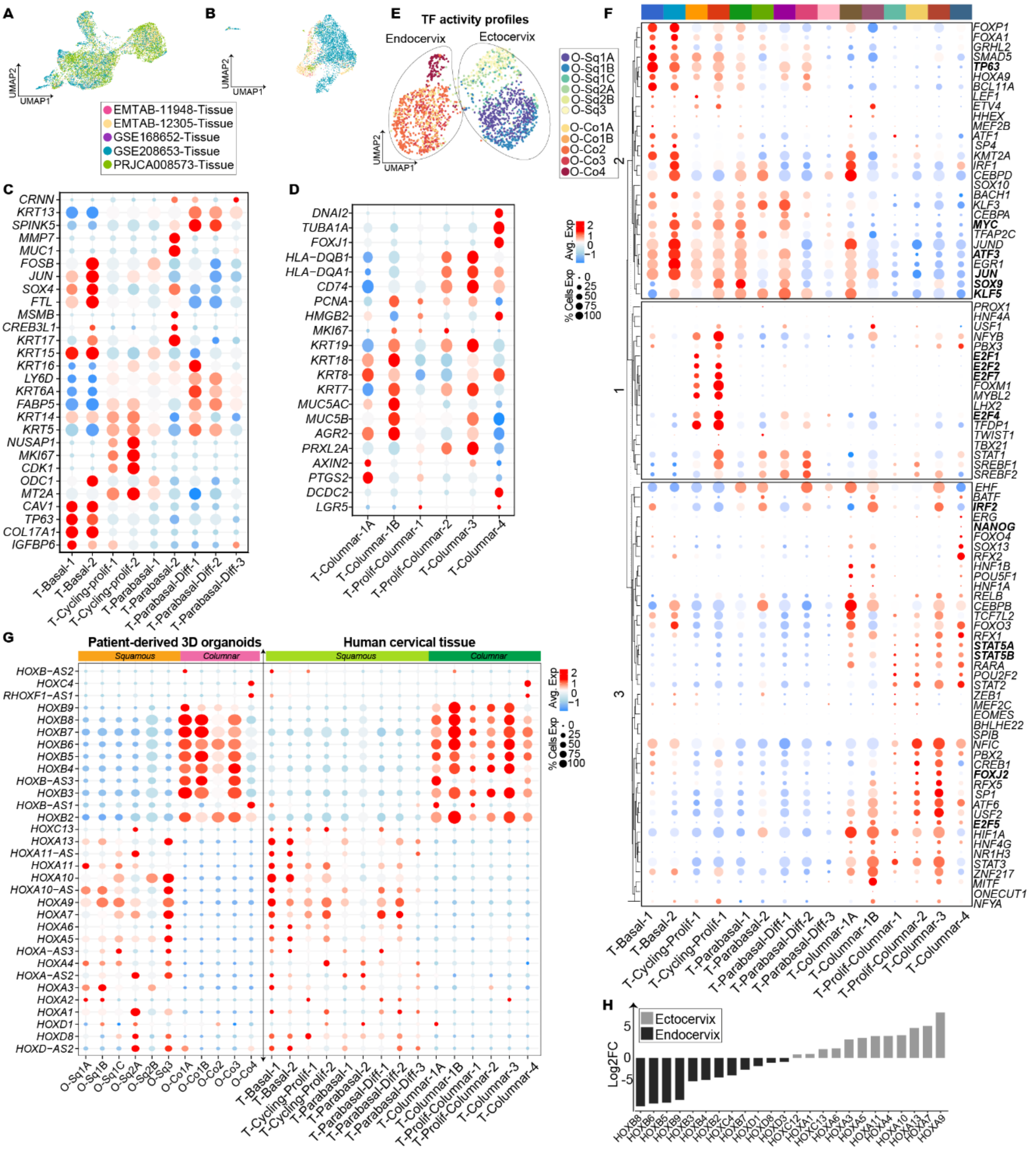


**Figure S3: Comparative single-cell analysis of squamous and columnar epithelial subsets in cervical organoids and native tissue.**

**A-B)** UMAP projections showing clustering for squamous (A) and columnar (B) epithelial cells from cervical tissue scRNA-seq datasets; cells colored by their dataset of origin. **C-D)** Dot plot showing the relative expression of canonical squamous (C) and columnar (D) markers used for annotating epithelial subclusters in tissue; circle size represents the percentage of cells expressing a particular gene, the color bar indicates the intensity of scaled mean expression levels ranging from high (red) to low (blue). **E)** UMAP visualization of epithelial cell clustering

solely based on their transcription factors (TF) activity profiles; each dot represents a single cell, colored by cluster annotation. **F)** Clustered dot plot showing the expression of selected TFs in tissue scRNA-seq data that were initially identified as differentially expressed between squamous and columnar epithelial subsets in organoids, confirming conserved expression patterns across both models. Circle size and color bar scaling as in (C, D). **G)** Dot plot from scRNA-seq data showing the expression levels of the development-related HOX genes in both ecto- and endocervical epithelial subclusters; circle size and color bar scaling as in (C, D). **H)** Bar graph showing DEGs of HOX family between ecto- and endocervix tissue based on microarray data.

**Figure S4**

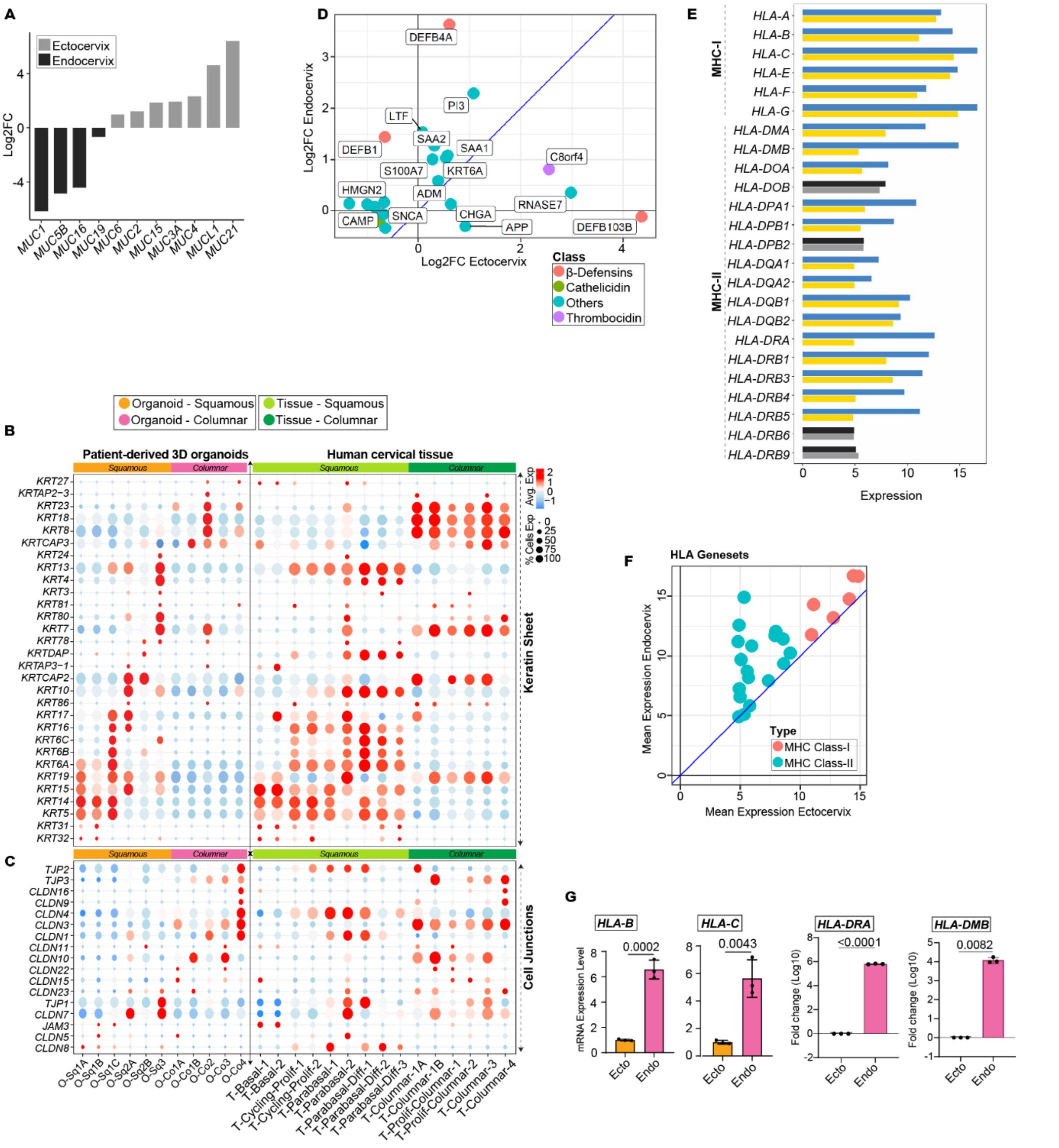


**Figure S4. Microarray and scRNA-seq profiling reveals region-specific expression of innate defense markers in the uterine cervix.**

**A)** Bar plot showing differential mucin gene expression between ecto- and endocervix tissue based on microarray data; bars are color-coded by tissue type. **B-C)** Dot plot highlighting the relative expression of epithelial barrier-associated genes including keratins (B) and gap junction proteins (C) across epithelial subtypes from organoid and tissue datasets. Circle size indicates the proportion of cells expressing each gene; color intensity reflects scaled mean expression from high (red) to low (blue). **D)** Scatter plot showing differential expression of

antimicrobial peptides (AMPs) between ecto- and endocervix based on log2 fold change from microarray analysis; dots are colored according to AMP class. **E)** Bar graph visualization of average expression levels of MHC genes that were differentially regulated between ecto- (yellow) and endocervix (blue) based on microarray analysis; non-differentially regulated genes were colored in gray and black for ecto and endo respectively. **F)** Scatterplot visualization of differentially expressed major histocompatibility complex (MHC) genes between ecto- and endocervical organoids. **G)** qRT-PCR analysis of MHC class I (*HLA-B*, *HLA-C*) and class II (*HLA-DRA*, *HLA-DMB*) gene expression in ecto- and endocervical organoids. Data presented as mean ± s.d. from three technical replicates, normalized to the ectocervix control, with p-values calculated using Student's t-test.

## Figure S5

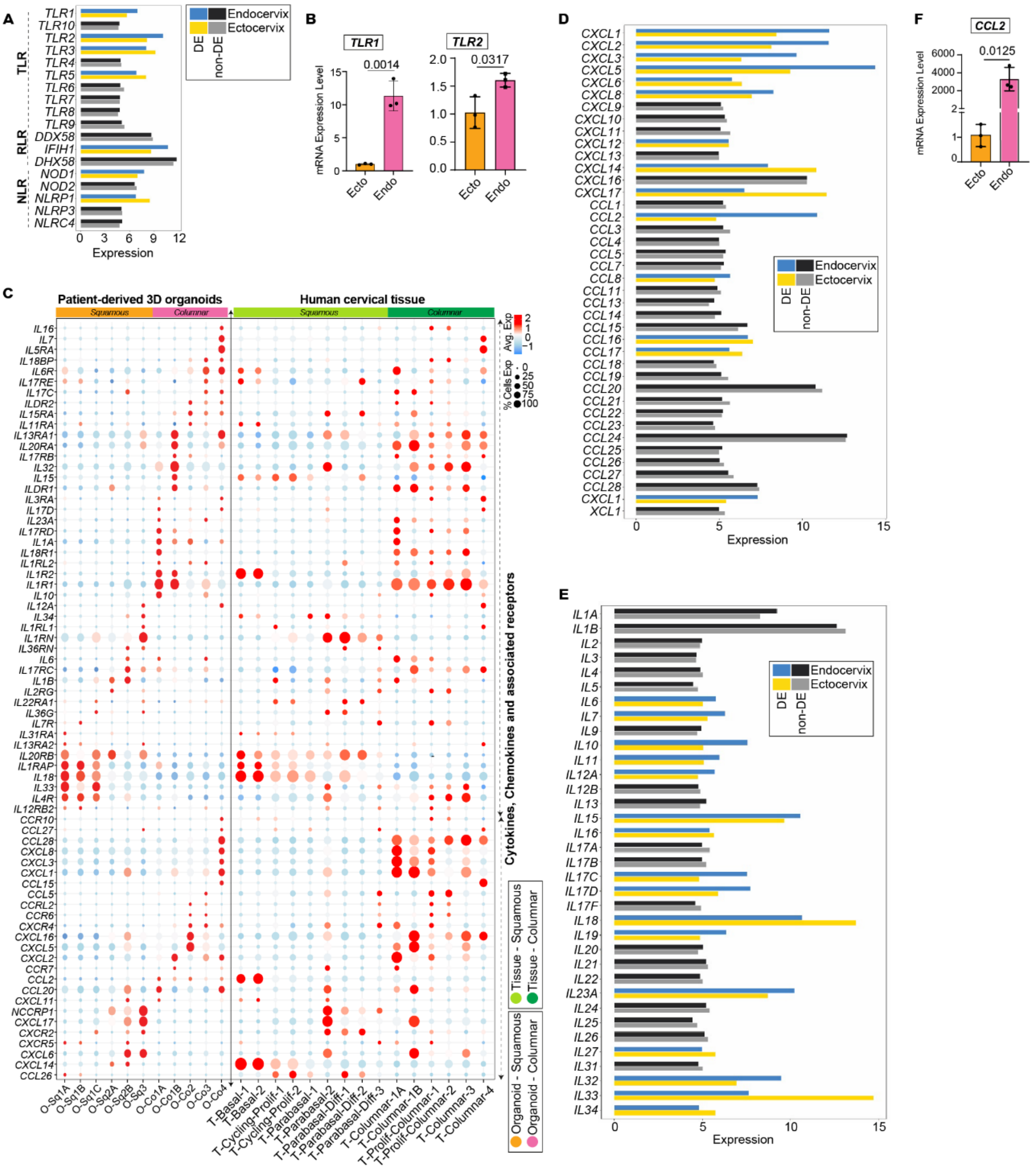


**Figure S5. Cytokine and chemokine gene expression modules across squamous and columnar epithelia of organoids and tissue.**

**A)** Bar graph visualization of average expression levels of PRRs, including Toll-like receptors (TLR), RIG-I-like receptors (RLR), and Nod-like receptors (NLR) genes that were differentially regulated between ecto- (yellow) and endocervix (blue) based on microarray analysis; non-differentially regulated genes were colored in gray and black for ecto and endo respectively. **B)** qRT-PCR validation of *TLR1*, *TLR2* expression in ecto- and endocervix organoids. Data presented as mean ± s.d. from three technical replicates, normalized to the ectocervix control, with p-values calculated using Student's t-test. **C)** Dot plot depicting the relative expression of

cytokine and chemokine genes across both organoid and tissue epithelial subsets; circle size represents the percentage of cells expressing a particular gene, while the color bar indicates the intensity of scaled mean expression levels from high (red) to low (blue). **D-E)** Bar plots from microarray analysis showing the mean expression values of cytokines and chemokines that were differentially regulated between ecto- (yellow) and endocervix (blue); non-differentially regulated genes are colored in gray (ecto) and black (endo). **F)** qRT-PCR analysis of *CCL2* expression in the ecto- and endocervical organoids. Data represent mean ± s.d. from three technical replicates, normalized to ectocervix control, and p-values were calculated using Student's t-test.

## Figure S6

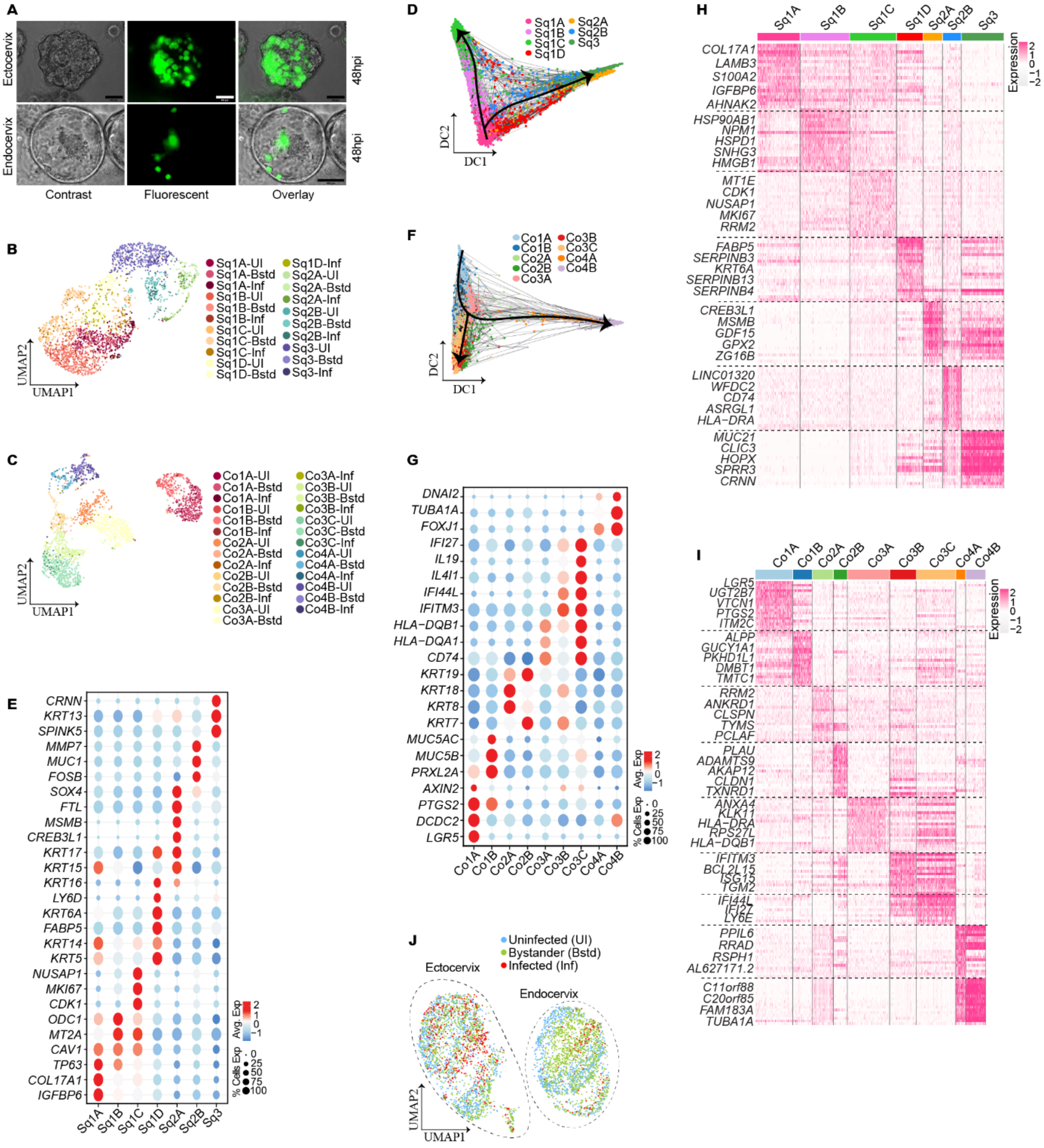


**Figure S6. Comprehensive analysis of squamous and columnar epithelial subsets in cervical organoids following Chlamydia infection.**

**A)** Bright field and fluorescent microscopy images of the ectocervical (upper panel) and endocervical (lower panel) organoids infected with Chlamydia-*GFP* showing inclusion bodies (green) at 48hpi. **B-C)** UMAPs of squamous (B) and columnar (C) epithelial subsets from ecto- and endocervical organoids colored by subcluster, with information on infection status. **D)** DM

plot depicting the developmental trajectories associated with squamous epithelia across all infection conditions in the ectocervix; each dot represents a single cell, colored by subcluster. **E)** Dot plot showing expression of canonical marker genes used to define squamous epithelial subclusters; circle size represents the percentage of cells expressing a specific gene, and the color bar indicates the intensity of scaled mean expression levels ranging from high (red) to low (blue). **F)** DM showing differentiation of endocervical columnar cells from all infection conditions; cells colored by subcluster. **G)** Dot plot showing columnar epithelial subtype-specific marker gene expression levels, with circle size and color bar scaling similar to (c). **H-I)** Heatmap displaying top 5 DEGs across identified ectocervical (H) and endocervical (I) subclusters post-infection; columns represent single cells grouped as clusters, color bar denotes the expression range from high (pink) to low (grey). **J)** UMAP plot showing TF activity-based clustering of cells from both ecto- and endocervix following *Chlamydia* infection; each dot represents a single cell, colored by infection status.

# Figure S7

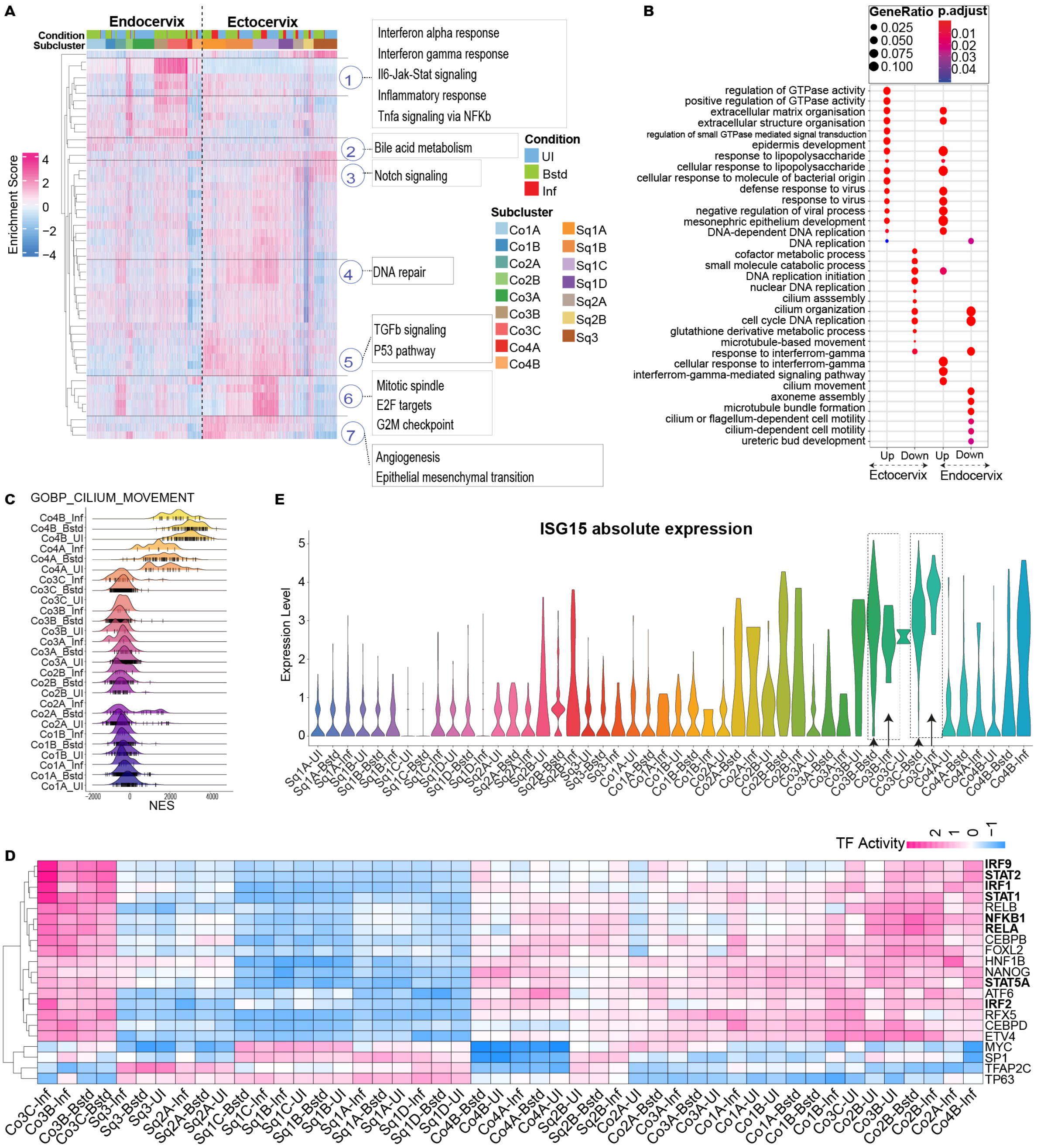


**Figure S7. Pathway-level and transcriptional regulatory landscape of cervical epithelial subsets in response to Chlamydia infection.**

**A)** Heatmap showing hallmark pathway enrichment scores across epithelial subtypes and infection conditions. Columns represent individual cells color-coded by tissue and infection status. **B)** Dot plot visualization of top 10 enriched GO terms for biological processes up- or down-regulated upon infection in ecto- and endocervix; circle diameter represents the gene

ratio within a group, fill color indicates adjusted P-value. **C)** Ridge plot showing distribution of normalized enrichment scores for GO term cilium movement across endocervical epithelial cells following infection at the subcluster level. **D)** Heatmap of TFs with highly variable activities across ecto- and endocervical samples and infection states, with subcluster annotations. **E)** Violin plot showing absolute expression levels of *ISG15* across ecto- and endocervical epithelial subclusters following Chlamydia infection.

## Figure S8

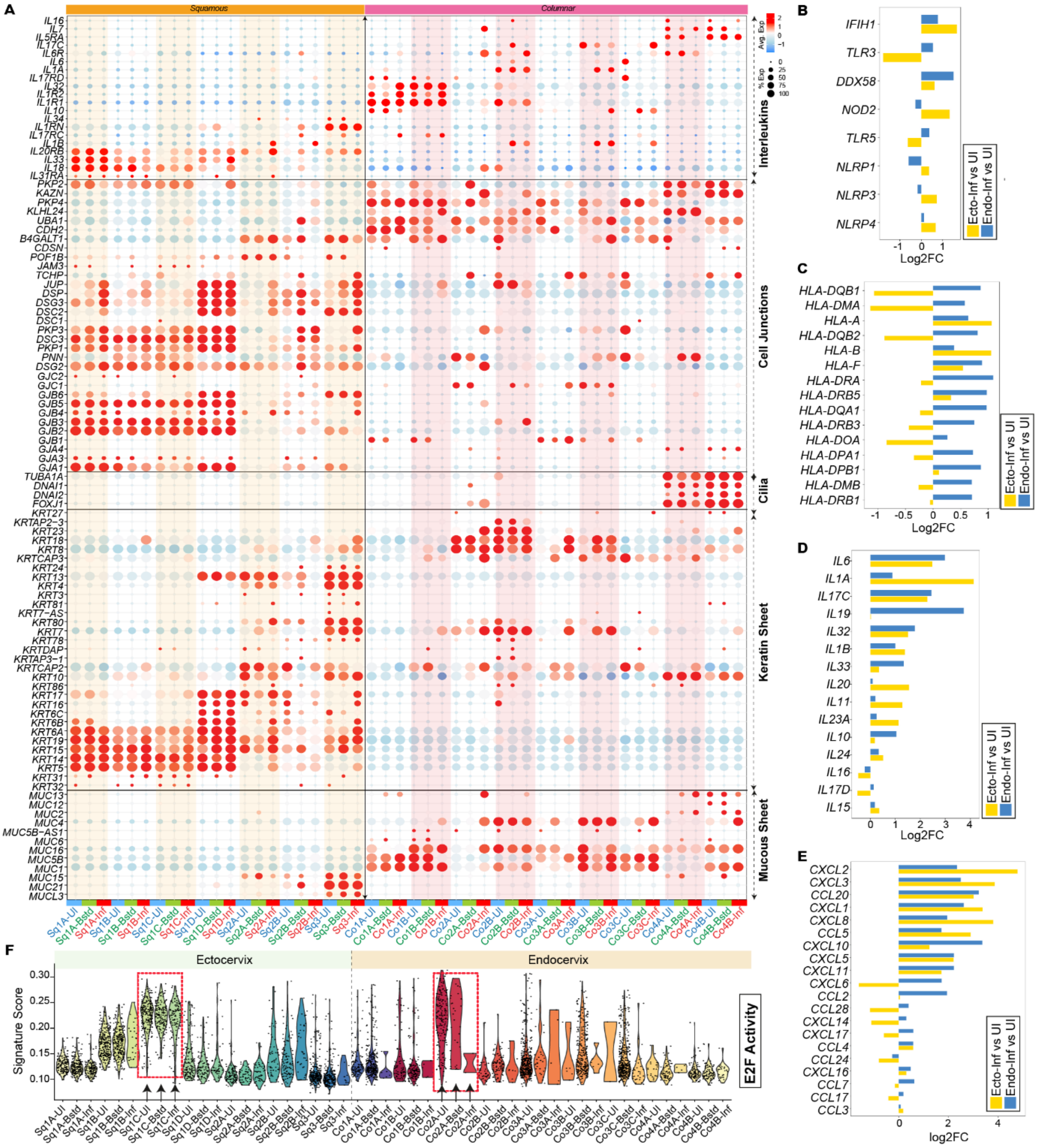

**Figure S8. Altered innate immune and proliferative gene expression patterns in the cervical epithelia following Chlamydia infection.**

**A)** Dot plot highlighting the expression of genes associated with physical and chemical mucosal defenses, including mucins, keratins, cilia, gap junctions, and interleukins across cervical epithelial subsets; circle size represents the percentage of cells expressing a particular gene, while the color bar indicates the intensity of scaled mean expression levels from high (red) to low (blue). Cluster labels are colored by infection status: blue for uninfected

(UI), green for bystander (Bstd), and red for infected (Inf). **B-E)** Bar plots displaying the differentially expressed genes related to PRR (B), MHC (C), cytokines and chemokines (D-E) comparing Chlamydia-infected versus uninfected samples in ecto- (yellow) and endocervix (blue), based on microarray analysis. **F)** Violin plot showing E2F transcription factor activity scores across ecto- and endocervical epithelial subtypes under uninfected, bystander and infected conditions.

## Figure S9

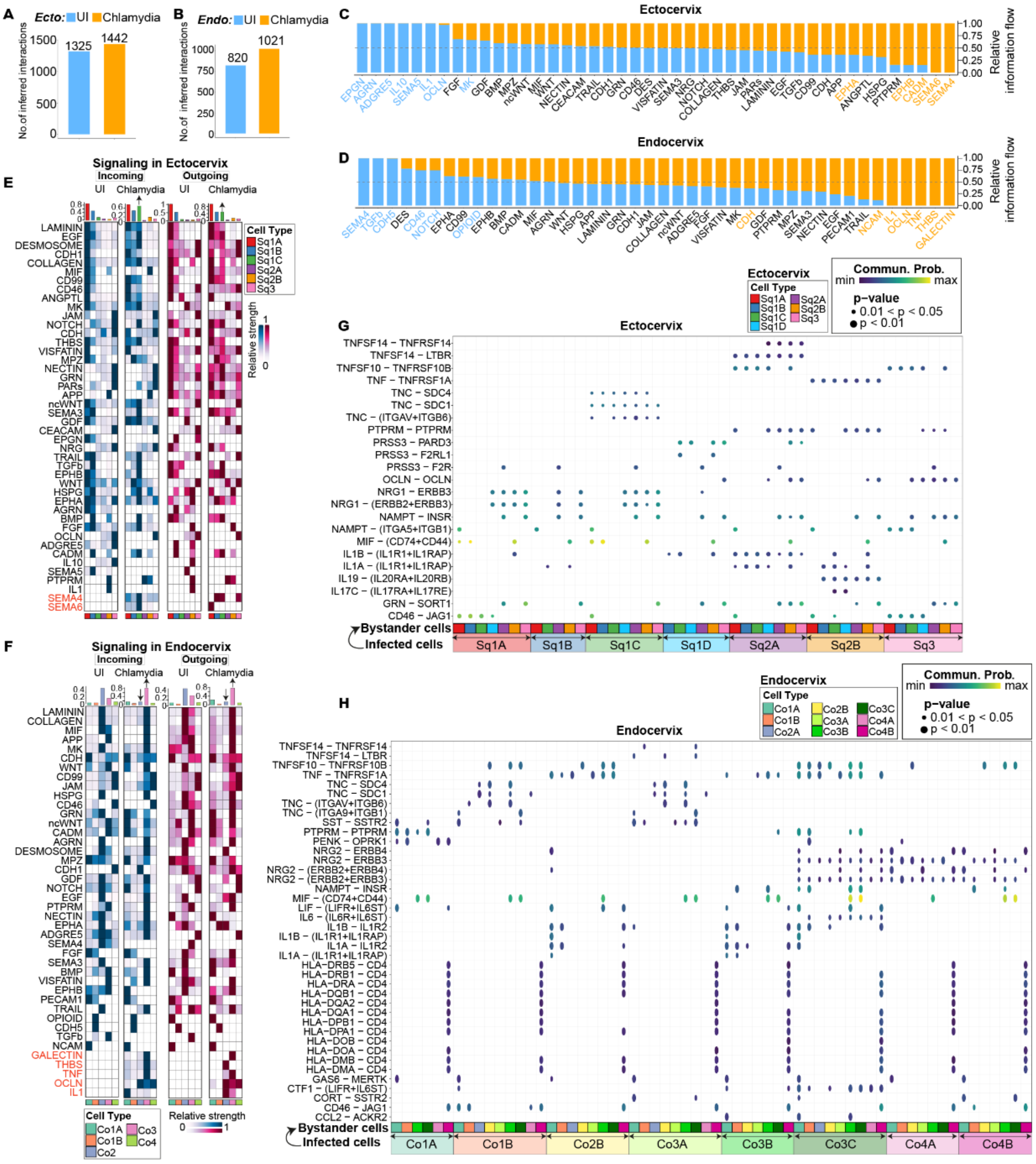

**Figure S9. *Chlamydia* infection remodels epithelial communication networks and signaling pathways in the uterine cervix.**

**A-B)** Bar plot showing the total number of interactions involved in the inferred cell-cell communication networks in ecto- (A) and endocervix (B) during steady state and infection; bars are colored by infection status, with uninfected in blue and infected in orange. **C-D)** Stacked bar graphs ranking significant signaling pathways based on differences in overall information flow/interaction strength within inferred networks between uninfected and infected conditions in ecto- (C) and endocervix (D); signaling pathways are colored in blue (enriched in steady state), orange (enriched in infection), or black (shared). **E-F)** Heatmap highlighting the changes in incoming and outgoing signaling patterns between steady state and infection

condition in ecto- (E) and endocervical (F) epithelial subsets; scale bar denotes the relative (row-scaled values) signaling strength of a pathway across cell types. colored bar plot on top shows the total signaling strength of each cell type by summarizing all pathways in the heatmap. **G-H)** Dot plot illustrating significant immunomodulatory interactions initiated by infected cells targeting bystander cells in ecto- (G) and endocervix (H); dot color and size reflect the communication probability and *p*-values; *p*-values were computed using a one-sided permutation test.

**Description of supplementary data tables**

**Table S1.** Differentially expressed genes between ecto- and endocervical organoids, related to figure S1B.

**Table S2.** Differentially regulated genes across squamous epithelial subclusters of ectocervix, related to figure S1F.

**Table S3.** Differentially regulated genes across columnar epithelial subtypes of endocervix, related to figure S1J.

**Table S4.** Activity scores of variable transcription factors (TF) across ecto- and endocervical epithelial cell clusters, related to figure 2H.

**Table S5.** Differentially regulated genes across squamous epithelial subclusters identified upon Chlamydia infection in ectocervix, related to figure S6H.

**Table S6.** Differentially regulated genes across columnar epithelial subclusters identified upon Chlamydia infection in endocervix related to figure S6I.

**Table S7.** Activity scores of variable transcription factors (TF) across ecto- and endocervical uninfected, infected and bystander epithelia, related to figure 4I.

**Table S8.** Gene set enrichment scores of ecto- and endocervical epithelial subtypes for hallmark pathways at different infection conditions, related to figure S7A.

**Table S9.** Activity scores of variable transcription factors (TF) across ecto- and endocervical epithelial subsets during steady state and infection conditions, related to figure S7D.

**Table S10.** Relative interaction strength scores for incoming signaling pathways across infected ectocervical epithelial cell types, related to figure S9E.

**Table S11.** Relative interaction strength scores for outgoing signaling pathways across infected endocervical epithelial cell types, related to figure S9F.

**Data S1**. Quantitative source data are provided for the following figures: Fig. 5B, 5C, 5D, 5E, S4G, S5B, and S5F in the accompanying Source Data file.
